# The roles of the acetyltransferase domains of the chromatin regulators KAT6A and KAT6B *in vivo*

**DOI:** 10.1242/dev.205559

**Published:** 2026-06-18

**Authors:** Tim Thomas, Shezlie Malelang, Yuqing Yang, Maria I. Bergamasco, Andrew J. Kueh, Alexandra L. Garnham, Nishika Ranathunga, Gordon K. Smyth, Anne K. Voss

**Affiliations:** ^1^ The Walter and Eliza Hall Institute of Medical Research, Melbourne, VIC 3052, Australia; ^2^ Department of Medical Biology, University of Melbourne, Melbourne, VIC 3010, Australia; ^3^ School of Mathematics and Statistics, University of Melbourne, Parkville, VIC 3010, Australia

**Keywords:** Histone acetyltransferase, KAT6A, KAT6B, Skeleton, Heart development, Hematopoietic stem cells, MOZ, MORF, QKF, Mouse

## Abstract

KAT6A (MOZ) and KAT6B (QKF/MORF) are related histone lysine acetyltransferases (KATs) that have a high degree of functional redundancy during development. In the absence of KAT6A, embryos undergo an anterior homeotic transformation of the axial skeleton, develop an interrupted aortic arch, have ventricular septal defects and fail to form definitive hematopoietic stem cells. KAT6B has roles in brain, skeletal and hematopoietic system development. Because loss of KAT6A leads to highly penetrant phenotypes, this allows us to determine whether the acetylation function is essential for all activities. We show that loss of acetyltransferase activity did not phenocopy the loss of the KAT6A protein in mice. Although mutation of the KAT domains of both KAT6A and KAT6B together increased the severity of phenotypes observed, these were milder than complete KAT6A loss of function. KAT domain mutants displayed ventricular septal defects and reduced (but not eliminated) hematopoietic stem cell activity. However, they did not display homeotic transformations or aortic arch defects, suggesting that, while acetylation is important for some functions, others can proceed without this activity. Accordingly, KAT6 proteins appear to have functions beyond acetylation.

## Teaser

**Highlighted Article:** KAT6A and KAT6B are epigenetic regulators and essential for normal development – if dysregulated they are potent oncogenes. This study shows that their function is not solely dependent on their enzymatic activity.

## INTRODUCTION

The regulation of gene expression is dependent on the action of large protein complexes that regulate access to chromatin. These protein complexes typically contain enzyme subunits that covalently modify histones, for example lysine acetyltransferases (KATs) (Huang et al., 2016; Voss and Thomas, 2009, 2018). The largest family of histone acetyltransferases in mammals is the MYST family (Sheikh and Akhtar, 2019; Thomas and Voss, 2007), which includes KAT6A (MOZ, monocytic leukemia zinc finger protein), an oncogene identified in an aggressive form of acute myeloid leukemia (Borrow et al., 1996). Studies of post translational modifications of histones have led to a ‘covalent modification’-centric view of how these epigenetic regulators function in transcription (Berger, 2007; Carlson and Glass, 2014; Kouzarides, 2000; Li et al., 2007; Strahl and Allis, 2000). In this model, protein-protein interaction domains (readers) within the complex serve primarily to direct the complex to sites in the chromatin, whereupon the enzyme subunit (writer) creates a covalent modification that changes the chromatin state until it is removed by an opposing enzyme (eraser). How readers, writers and erasers function to regulate gene expression is important to understand the pathogenesis of disease, in particular cancer (Zhao et al., 2021). Nevertheless, emphasis on the covalent modification of histones may obscure other activities of these complexes that do not require enzymatic activity.

The KAT6A complex typically includes ING5, BRPF1 and MEAF6 subunits (Doyon et al., 2006; Mousavi and Yang, 2025; Ullah et al., 2008). BRPF1 is a large multidomain protein (Mousavi and Yang, 2025) that can bind acetylated histones via its bromodomain (Obi et al., 2020; Poplawski et al., 2014) and associate with DNA in chromatin via its PZP domain (Klein et al., 2020). ING5 can bind methylated histone 3 lysine 4 (H3K4) (Ormaza et al., 2019). KAT6A has a double PHD finger, can bind acetylated H3K14 contingent on the modification status of surrounding residues (Ali et al., 2012; Dreveny et al., 2014; Qiu et al., 2012) and contains N-terminal wing helix domains that can bind un-methylated CpG islands (Becht et al., 2023; Weber et al., 2023). The related protein KAT6B (Querkopf, MORF) has an identical domain structure to KAT6A (Ullah et al., 2008) and can completely rescue loss of KAT6A when overexpressed (Bergamasco et al., 2025c). KAT6A and KAT6B function in large complexes containing multiple ‘reader’ domains that can specifically recognize different histone modifications, and so different chromatin states, but with a single enzymatic acetyltransferase domain. This raises the question of whether the chromatin recognition domains function only to direct the acetylation domain to sites within chromatin, or whether the complex has activity independent of acetylation.

Chromatin remodeling is crucial for embryonic development (Chen and Dent, 2014) and so it is not surprising that disruption of KAT6A or KAT6B function leads to severe developmental defects. Mutation in KAT6A leads to Arboleda-Tham cognitive impairment syndrome in children (Arboleda et al., 2015; Tham et al., 2015). Mutations in KAT6B result in several syndromes that have related phenotypes (Clayton-Smith et al., 2011; Kraft et al., 2011; Simpson et al., 2012), principally Genitopatellar syndrome (Simpson et al., 2012) and Say-Barber-Biesecker-Young-Simpson variant of Ohdo syndrome (Clayton-Smith et al., 2011).

KAT6A has key roles in multiple aspects of embryonic development. Heterozygous loss of *Kat6a* in mice causes learning and memory defects that are partially improved by elevating histone acetylation (Eccles et al., 2026). KAT6A regulates Hox gene expression and loss of *Kat6a* results in a complete homeotic transformation of the axial skeleton (Voss et al., 2009). Notably, KAT6A directly opposes the action of BMI1 (PCGF4), a polycomb group protein that represses Hox gene expression (Sheikh et al., 2015a). KAT6A mutant mice phenocopy DiGeorge syndrome, resulting in an interrupted aortic arch type B and ventricular septal defects (VSDs), as well as cranio-facial abnormalities (Vanyai et al., 2019, 2015; Voss et al., 2009, 2012). Loss of KAT6A function results in the failure of definitive hematopoietic stem cells (HSCs) to develop and be maintained in adulthood (Katsumoto et al., 2006; Sheikh et al., 2017, 2016; Thomas et al., 2006). Interestingly, abrogating the function of the acetyltransferase domain does not completely remove HSC activity, suggesting that some functions may not entirely depend on the lysine acetyltransferase activity (Perez-Campo et al., 2009).

KAT6B acts primarily in development of the central nervous system (Merson et al., 2006; Rietze et al., 2001; Sheikh et al., 2012; Thomas et al., 2000). KAT6B is required for normal functioning of adult neural stem cells (Merson et al., 2006). Reduced KAT6B function in heterozygous mice leads to learning and memory defects, which can be partially corrected by increasing histone acetylation levels (Bergamasco et al., 2024b). Conversely, overexpression of KAT6B leads to anxiety and aggression (Bergamasco et al., 2025b). KAT6B is also essential for bone development and full HSC activity (Bergamasco et al., 2024a, 2025a).

Deregulation of KAT6A and KAT6B can lead to cancer (Thomas and Voss, 2007; Wiesel-Motiuk and Assaraf, 2020; Zheng et al., 2025). Conversely, reduction of KAT6A in a mouse model of MYC-driven lymphoma delays onset of disease (Sheikh et al., 2015b). Due to the importance of KAT6 proteins in oncogenesis, we developed inhibitors targeting both KAT6A and KAT6B that reduce KAT activity to background levels (Baell et al., 2018). *In vitro*, these compounds induce mouse embryonic fibroblasts (MEFs) to become senescent, which is also seen in MEFs lacking KAT6A (Sharma et al., 2023; Sheikh et al., 2015c), and arrest progression of lymphoma (Baell et al., 2018). KAT6 drugs are now in clinical trials for the treatment of breast cancer (Mukohara et al., 2024).

As KAT6A and KAT6B have essential roles in embryonic development and are clinically relevant drug targets, we examined the relative importance of KAT activity for overall protein function. We generated point mutants in the KAT domain that prevent acetylation of substrates and studied the effect of these on KAT6A function and on the combined function of KAT6A and KAT6B. We concentrated this analysis on hematopoiesis, axial skeletal development and heart development, as KAT6A is essential for these structures, and phenotypic anomalies in these organ systems are highly penetrant in *Kat6a* null mutant mice. We found that KAT activity is essential for normal hematopoiesis and for normal heart development. However, there was no homeotic transformation of the axial skeleton or defects in the aortic arch development, although some comparatively minor defects in bone growth were present. While hematopoiesis was severely affected, stem cell activity was not eliminated. These results suggest that the essential roles of KAT6 proteins and their associated complex are not limited to acetylation.

## RESULTS

### Effects of point mutations in the acetyltransferase domain of KAT6A and KAT6B on histone acetylation, development and survival *in vivo*

**Fig. 1.**
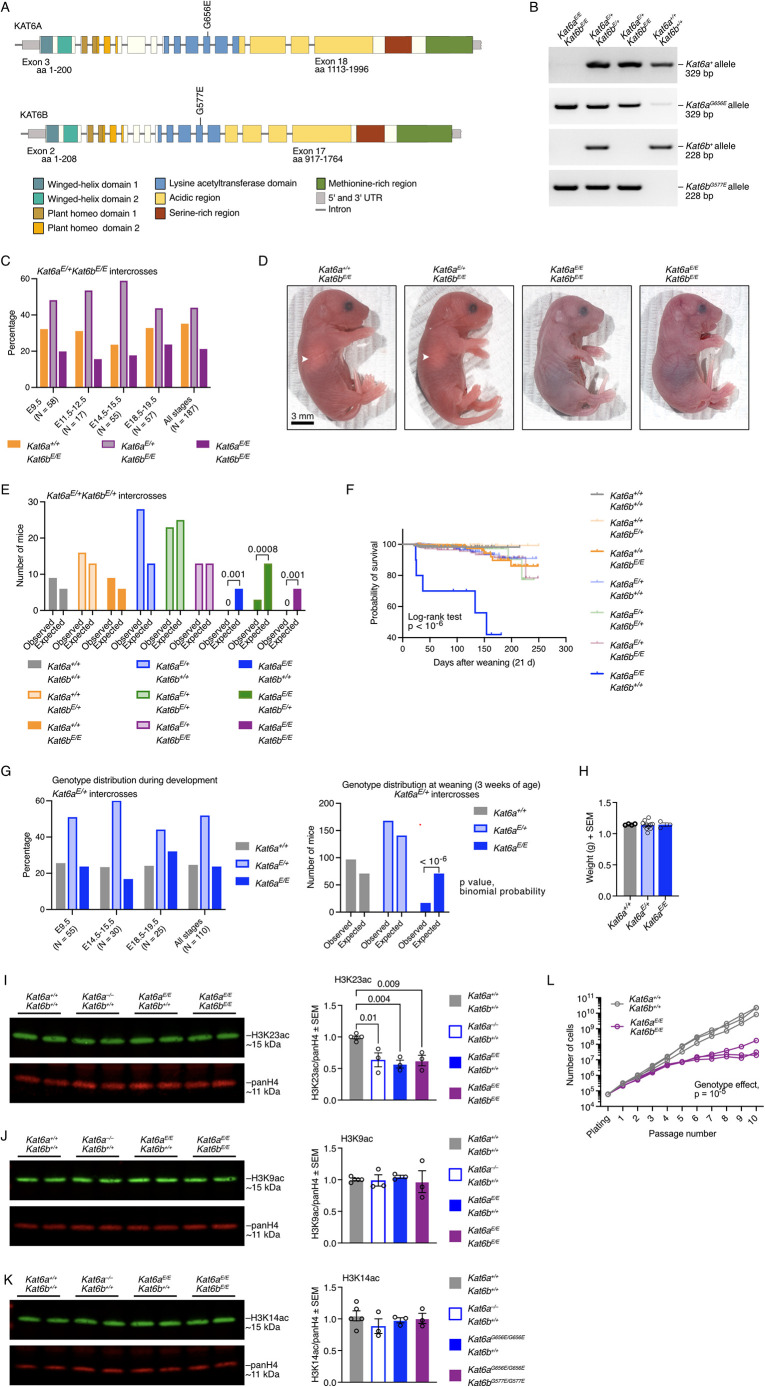
**Combined loss of acetylation activity of KAT6A and KAT6B leads to perinatal lethality.** (A) Exon and protein domain structure of mouse KAT6A and KAT6B and the position of the *Kat6a^G658E^* and *Kat6b^G577E^* point mutations. Protein domains are indicated by colors. Introns are not to scale. (B) Representative PCR genotyping gel of *Kat6a^G658E^* (*Kat6a^E^*) and *Kat6b^G577E^* (*Kat6b^E^*) point mutant alleles, showing mutant (amplicon present only in mutant reaction), heterozygous (amplicon present in mutant and WT reaction) and WT (amplicon present only in WT reaction) results. (C) Genotype distribution of offspring of *Kat6a^E/+^*;*Kat6b^E/E^*×*Kat6a^E/+^*;*Kat6b^E/E^* matings. *Kat6a^E/E^;Kat6b^E/E^* fetuses were present at birth (*n*=187 embryos and fetuses). (D) Representative images of *Kat6a^E/E^;Kat6b^E/E^* pups postpartum compared to *Kat6b^E/E^* and *Kat6a^E/+^;Kat6b^E/E^* littermates, recovered at E19.25/P1 by caesarean section. All pups shown here were alive as indicated by movements of the body. *Kat6a^E/E^;Kat6b^E/E^* pups, but not pups of any other genotype, delayed or failed to inflate their lungs, oxygenate their blood and appeared ‘blue’ rather than pink. Arrowheads indicate lighter colored area of the inflated lungs in littermates. Scale bar: 3 mm. (E) Expected compared to observed offspring genotype distribution at weaning of *Kat6a^E/+^;Kat6b^E/+^*×*Kat6a^E/+^;Kat6b^E/+^* matings (*n*=101 mice at weaning). (F) Kaplan-Myer survival graph of mice from weaning to 250 days (*n*=1314 mice). (G) Genotype distribution of offspring of *Kat6a^E/+^*×*Kat6a^E/+^* matings pre- and post-partum (left and right, respectively). Left, *n*=110 embryos and fetuses. Right, *n*=282 3-week-old mice. (H) Body weight of E18.5 fetuses of *Kat6a^E/+^* × *Kat6a^E/+^* matings (*n*=4 *Kat6a^+/+^;Kat6b^+/+^*, 11 *Kat6a^E/+^* and 3 *Kat6a^E/E^* fetuses). (I-K) Western blot detection and quantitation relative to pan-H4 of H3K23ac (I), H3K9ac (J) and H3K14ac (K) in comparing whole E9.5 embryos (*n*=3-5 embryos per genotype for the quantitation, with 2 per genotype shown in the western blots). (L) Cumulative growth curves of *Kat6a^E/E^;Kat6b^E/E^* mouse embryonic fibroblasts compared to wild type. Data are presented as percentage of animals (C,G left), number of animals (E,G right), percentage survival (F), mean±s.e.m. (H-K) and number of cells (L). Each circle represents one mouse (H-K). Each point is the average of each triplicate culture of cells isolated from one embryo (L). Data were analyzed by calculating binomial probability of a difference to the expected value (C,E,G) or using the log-rank Mantel-Cox test (F), one-way ANOVA with Tukey's multiple comparisons test (H-K) or two-way ANOVA with Šídák's multiple comparisons test (L).

To determine the role of acetylation in the function of KAT6A and KAT6B we generated point mutations in the KAT domain of these epigenetic regulators using CRISPR. A single amino acid change G656E was created in KAT6A and a G577E mutation was created in KAT6B (Fig. 1A; Table S1). These mutations are equivalent to previously reported mutations in MYST family members and have been shown to reduce acetyltransferase activity to background levels (Akhtar and Becker, 2000; Deguchi et al., 2003; Lv et al., 2017; Perez-Campo et al., 2009; Takechi and Nakayama, 1999). These changes alter homologous, highly conserved glycine residues that are part of the acetyl-CoA binding domain and are compatible with *in vivo* production of a full-length protein. Genotyping was performed using two separate PCR reactions (Table S1) to determine the presence of the wild-type (WT) or mutant allele (Fig. 1B).

Inter-crossing mice homozygous for the *Kat6b^G577E^* allele (abbreviated *Kat6b^E^*) and heterozygous for the *Kat6a^G656E^* allele (*Kat6a^E^*) showed that double homozygosity for the point mutations in the KAT domains, *Kat6a^E/E^;Kat6b^E/E^*, was compatible with development to term (Fig. 1C; *n*=187). Embryos homozygous for *Kat6b^E^* point mutant alleles appeared to be outwardly normal. Among pups recovered by caesarean section at gestational day (E)19.25 (the morning when birth was expected), pups of all genotypes were alive. However, *Kat6a^E/E^;Kat6b^E/E^* pups showed a marked delay in lung inflation and change in skin color to a healthy pink, indicating a delay in oxygenating blood (Fig. 1D). No *Kat6a^E/E^;Kat6b^E/E^* pup had milk in their stomach, indicating that they did not suckle successfully. No living double homozygous pups were found after postnatal day (P)1. Thus, *Kat6a^E/E^;Kat6b^E/E^* pups developed to birth – significantly further than *Kat6a^–/–^;Kat6b^gt/gt^* compound homozygous mutant embryos, which arrested in development at E9.0 (Fig. S1).

At 3 weeks of age, when pups were weaned, no *Kat6a^E/E^* were present among offspring of *Kat6a^E/+^*;*Kat6b^E/+^*×*Kat6a^E/+^*;*Kat6b^E/+^* matings (Fig. 1E). Three *Kat6a^E/E^*;*Kat6b^E/+^* mice survived to weaning (Fig. 1E). A single *Kat6a^E/E^*;*Kat6b^E/+^* mouse survived to adulthood. Reduction in the number of mice observed versus expected occurred in genotypes that included *Kat6a^E^* homozygosity, but not those with merely *Kat6b^E/E^* homozygosity, suggesting that point mutation of the KAT domain of KAT6A was the primary cause of prenatal and perinatal losses.

Among offspring of *Kat6a^E/+^*;*Kat6b^+/+^*×*Kat6a^E/+^*;*Kat6b^+/+^* matings, ten *Kat6a^E/E^*;*Kat6b^+/+^* pups out of 101 survived weaning but exhibited reduced vitality, with only two surviving more than 150 days after weaning (Fig. 1F,G). There was no effect of the *Kat6a^E/E^*;*Kat6b^+/+^* genotype on survival to term (Fig. 1G) or body weight at E18.5 (Fig. 1H).

Loss of KAT6A function leads to a global reduction in H3K23ac (Bergamasco et al., 2025c; Lv et al., 2017). We performed western blots and densitometry (Fig. 1I-K) to determine the functional effect of point mutations. We observed a reduction in H3K23ac levels in whole *Kat6a^E/E^* (*P*=0.004) and *Kat6a^E/E^;Kat6b^E/E^* (*P*=0.009) embryos that was not statistically different to the reduction observed in *Kat6a^–/–^* (*P*=0.01; Fig. 1I) (all comparisons were with WT embryos). No change in the levels of H3K9ac or H3K14ac were observed (Fig. 1J,K). Inhibition of the acetyltransferase activity of KAT6A and KAT6B with inhibitors led to cell cycle arrest in mouse embryonic fibroblasts (Baell et al., 2018). As expected, fibroblasts derived from *Kat6a^E/E^;Kat6b^E/E^* E12.5 embryos underwent cell cycle arrest (*P*=0.000014; Fig. 1L), also demonstrating that point mutations impaired acetylation activity.

### Effects of point mutations compared to null mutations of *Kat6a* and *Kat6b* on gene expression in E9.5 embryos

We determined the effects of the point mutations on gene expression and, using RNA-sequencing, compared these with the effect of removing KAT6A and KAT6B function entirely. Whole WT, *Kat6a^–/–^*, *Kat6b^–/–^*, *Kat6a^E/E^*, *Kat6b^E/E^* and *Kat6a^E/E^;Kat6b^E/E^* E9.5 embryos were dissected then stage and sex-matched (Fig. S2) for RNA isolation and bulk RNA-sequencing analysis.

**Fig. 2.**
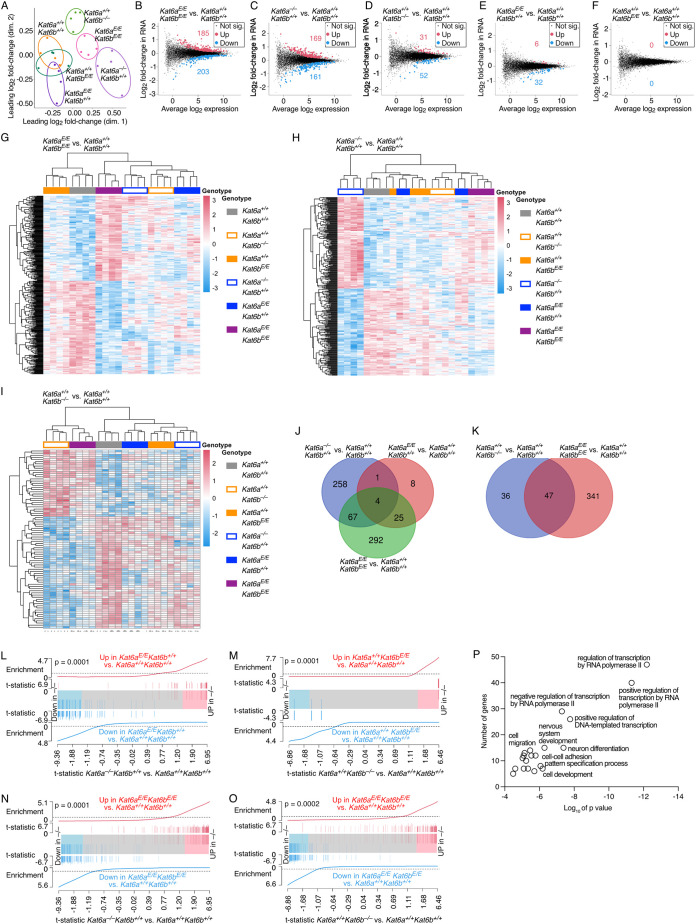
**Combined loss of acetylation activity of KAT6A and KAT6B causes gene expression changes intermediate between complete loss of each gene.** (A-P) For bulk RNA-sequencing, *n*=4 whole E9.5 male embryos per genotype were used. Data were analyzed as described in the ‘RNA-sequencing data analysis’ section. FDR<5% was considered significant. (A) Multidimensional scaling plot of the leading gene expression differences (dimension 1 and 2) between samples in pair-wise comparisons of embryos of all six genotypes. (B-F) M (log_2_ ratio) and A (mean average) plot of differentially expressed genes between genotypes as indicated. Upregulated (red) and downregulated genes (blue) are indicated in each MA plot. Genes not significantly changed indicated in black. (G-I) Heatmaps showing differentially expressed genes comparing genotypes as indicated. (J,K) Venn diagrams displaying the overlap in gene expression changes in the comparisons as indicated. (L-O) Barcode plots showing correlations between gene expression changes in different genotype comparisons as indicated. The null mutation of *Kat6a* or *Kat6b* and the combined points mutation of all four *Kat6* alleles had strong effects so that FDR of 0.05 was used. To enable comparison of the single gene points mutations of either *Kat6a* or *Kat6b* the cut-off was relaxed to *P*<0.001. (P) Top 20 Gene Ontology terms (biological process) associated with genes differentially expressed in *Kat6b^–/–^* versus WT embryos, but not in *Kat6a^E/E^;Kat6b^E/E^* versus WT embryos.

Multidimensional scaling revealed that embryos of the two null mutant genotypes (*Kat6a^–/–^* and *Kat6b^–/–^*) clustered within genotype and separated from each other and the WT embryos (Fig. 2A). The double point mutant embryos (*Kat6a^E/E^;Kat6b^E/E^*) clustered within genotype and were positioned between the *Kat6a^–/–^* and the *Kat6b^–/–^* embryos. *Kat6b^E/E^* and *Kat6a^E/E^* single point mutant embryos did not obviously segregate from WT embryos (Fig. 2A). Accordingly, the largest number of differentially expressed genes (FDR<0.05) compared to WT embryos was observed in *Kat6a^E/E^;Kat6b^E/E^* embryos (388 genes: 203 downregulated, 185 upregulated), followed by *Kat6a^–/–^*embryos (330 genes: 161 downregulated, 169 upregulated) and the *Kat6b^–/–^* (83 genes: 52 downregulated, 31 upregulated; Fig. 2B-D; Tables S2-S4). In contrast, the single point mutant embryos did not show many differentially expressed genes compared to WT embryos (Fig. 2E,F; Tables S5 and S6).

Unsupervised hierarchical clustering revealed that, with respect to the genes that were differentially expressed between double point mutant *Kat6a^E/E^;Kat6b^E/E^* embryos and WT embryos, the *Kat6b^E/E^* single point mutant embryos clustered with WT embryos, whereas both null mutant genotypes (*Kat6a^–/–^*, *Kat6b^–/–^*) and *Kat6a^E/E^* single point mutant embryos clustered with the double point mutant embryos (Fig. 2G).

For genes differentially expressed between *Kat6b^–/–^* and WT embryos, embryos of all other genotypes displayed effects that were intermediate between *Kat6b^–/–^* and WT embryos (Fig. 2H). With respect to genes differentially expressed between *Kat6b^–/–^* and WT embryos, *Kat6b^–/–^* embryos clustered with *Kat6a^E/E^;Kat6b^E/E^* embryos, but not with any other genotype (Fig. 2I).

Of the 330 genes that were differentially expressed in *Kat6a^–/–^* embryos, only five were also differentially expressed at FDR<0.05 in *Kat6a^E/E^* embryos (1.5%; Fig. 2J), while 22% (71/330) were also differentially expressed in *Kat6a^E/E^;Kat6b^E/E^* embryos (Fig. 2J). Of 83 genes that were differentially expressed in *Kat6b^–/–^* embryos, 57% (47 genes) were also differentially expressed in *Kat6a^E/E^;Kat6b^E/E^* embryos (Fig. 2K). *Kat6b^E/E^* embryos did not display genes differentially expressed at FDR<0.05. However, enrichment analyses suggested that the expression of genes was affected similarly by *Kat6a* or *Kat6b* KAT domain point mutation and null mutation, albeit with lower effect sizes (Fig. 2L-O).

Gene ontology analysis was used to identify the biological processes affected by mutations (Tables S7-S12). The processes affected by complete loss of KAT6A uniquely included regulation of transcription by RNA polymerase II, nervous system development and pattern specification process, among others (Fig. 2P; Table S8). Only nine processes were affected in *Kat6a^E/E^;Kat6b^E/E^* embryos but not in *Kat6a^–/–^;Kat6b^+/+^* embryos, suggesting that the biological effects of the loss of the KAT6A KAT activity were largely contained within the complete loss of KAT6A. In contrast, in the comparison between the effects of complete loss versus acetyltransferase loss of *Kat6b* the largest number of biological processes were affected in *Kat6a^E/E^;Kat6b^E/E^* embryos, but not in *Kat6b^–/–^* embryos (Tables S10-S12), suggesting a smaller contribution of the loss of KAT6B to the effects of the *Kat6a^E/E ;^Kat6b^E/E^* point mutations.

Taken together, our E9.5 embryo RNA-sequencing data show that a larger number of gene expression changes are observed as result of the total loss of KAT6A or KAT6B compared to each KAT domain point mutation. However, while loss of the histone acetyltransferase activity causes fewer changes in gene expression, enrichment analyses suggested that loss of the acetyltransferase activity affected similar genes in the same direction as complete loss of KAT6A or KAT6B, albeit with a smaller effect size. Our data suggest that lysine acetyltransferase activity is only one of the functions of KAT6A and KAT6B, with other functions contributing to the total effects of these proteins on gene expression.

### Heart development in fetuses carrying point mutations in KAT6A and KAT6B

**Fig. 3.**
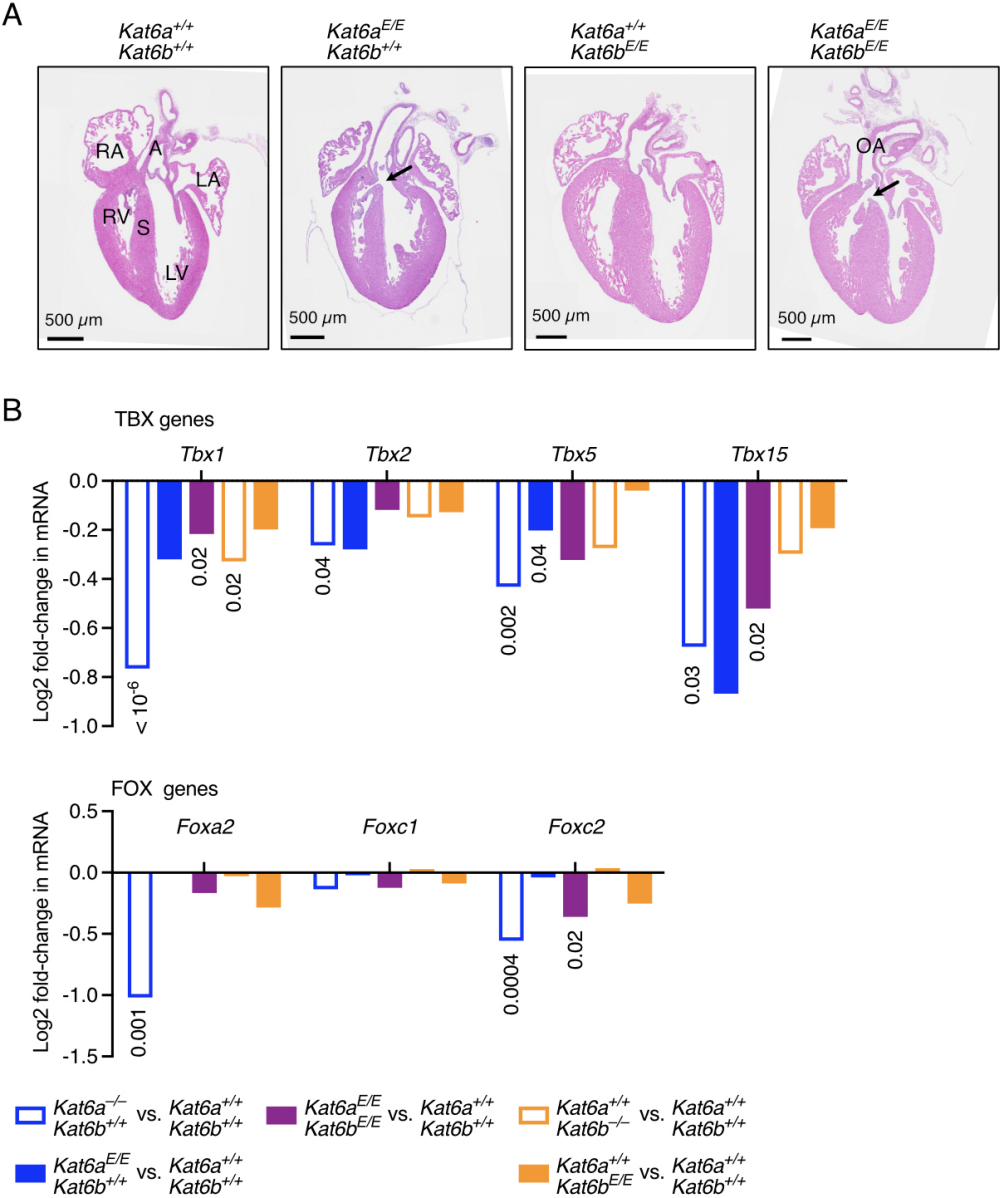
**Ventricular septal defects in *Kat6a^E/E^;Kat6b^E/E^* and *Kat6a^E/E^* mice.** (A) Representative images of H&E-stained serial sections of heart dissected from E19.25 fetuses of the genotypes indicated. Arrows indicate ventricular septal defects. A, aorta; LA, left atrium; LV, left ventricle; OA, overriding aorta; RA, right atrium; RV, right ventricle; S, interventricular septum. Scale bars: 500 μm. (B) Log_2_ fold-change in RNA levels of TBX and FOX genes. FDRs<0.05 are shown below the bars. *n*=4 whole E9.5 embryos per genotype were used for bulk RNA-sequencing. Data were analyzed as described in the ‘RNA-sequencing data analysis’ section. FDR<5% was considered significant.

We have previously shown that loss of KAT6A function leads to severe heart and aortic arch abnormalities, including VSDs and an interrupted aortic arch type B (Voss et al., 2012). We collected fetuses at E19.25 by caesarean section. Fetuses of the WT, *Kat6a^E/E^*, *Kat6b^E/E^* and *Kat6a^E/E^;Kat6b^E/E^* genotypes were collected for serial histological sectioning of the heart. All *Kat6a^E/E^;Kat6b^E/E^* fetuses examined (3/3), and 1/5 *Kat6a^E/E^* fetus examined had VSDs (Fig. 3A). No heart abnormalities were observed in WT or *Kat6b^E/E^* fetuses. No interrupted aortic arch was seen in *Kat6b^+/+^* (*n*=13), *Kat6a^E/+^* (*n*=11), *Kat6a^E/E^* (*n*=7), *Kat6b^E/E^* (*n*=14) or *Kat6a^E/E^;Kat6b^E/E^* (*n*=13) fetuses.

Of the diverse developmental phenotypic anomalies caused by *Kat6a* null mutation, only the VSDs were observed prominently in *Kat6a^E/E^;Kat6b^E/E^* point mutant fetuses. Gene expression changes associated with heart defects, previously found to be affected by loss of KAT6A (Voss et al., 2012), were present in both *Kat6a^–/–^* E9.5 and in *Kat6a^E/E^;Kat6b^E/E^* E9.5 embryos (Fig. 3B; bulk RNA-sequencing of whole E9.5 embryos). This included a notable reduction in *Tbx1*, *Tbx2*, *Tbx5* and *Tbx15* gene expression (FDR: <10^−6^-0.04) and *Foxa2*, *Foxc1* and *Foxc2* gene expression (FDR: 0.0004-0.04) in *Kat6a^–/–^* embryos (Fig. 3B). In *Kat6a^E/E^;Kat6b^E/E^* embryos some of these genes were reduced in expression, namely *Tbx1*, *Tbx5* and *Tbx15* (FDR: 0.02 to 0.04) and *Foxc2* (FDR: 0.02; Fig. 3B). A reduction in the difference in the *Kat6a^E/E^;Kat6b^E/E^* embryos compared to the *Kat6a^–/–^* embryos is associated with a reduced severity of the cardiovascular defects, namely the absence of an interrupted aortic arch in the *Kat6a^E/E^;Kat6b^E/E^* embryos. Note that an interrupted aortic arch occurs with 74% penetrance in embryos homozygous for the less penetrant *Kat6a^Δ^* allele, which produces low levels of KAT6A protein lacking the C-terminal domains (Voss et al., 2012).

### Axial skeleton morphology in fetuses carrying point mutations in KAT6A and KAT6B

**Fig. 4.**
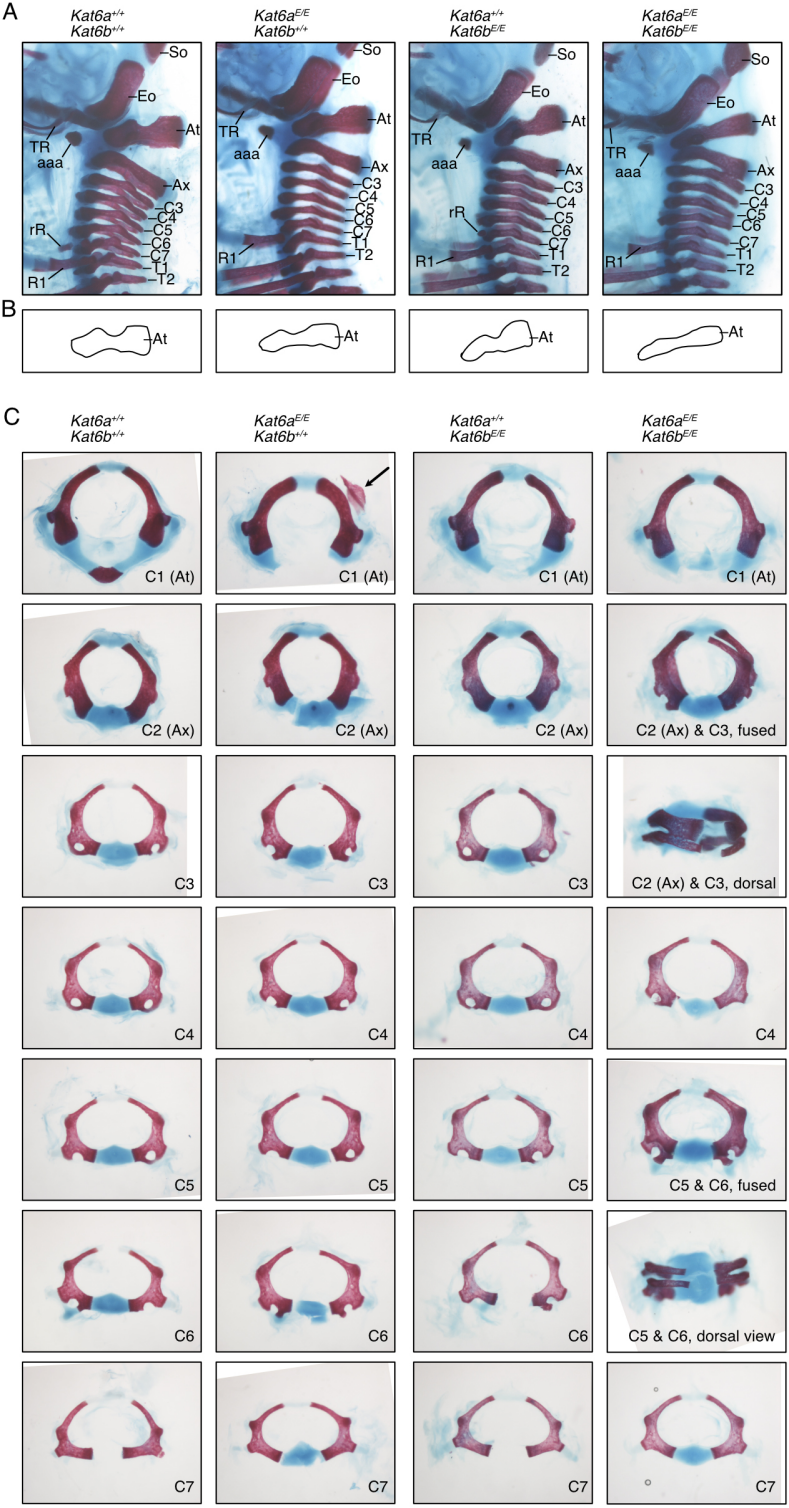
**Point mutation of the KAT domain of *Kat6a* and *Kat6b* does not lead to homeotic transformation of the axial skeleton.** (A) Representative images of skeletal preparations of E18.5 WT (*n*=3), *Kat6a^E/E^* (*n*=5), *Kat6b^E/E^* (*n*=5) and *Kat6a^E/E^;Kat6b^E/E^* (*n*=3) fetuses. (B) Line tracing of the C1 vertebra (atlas). Note that the *Kat6a^E/E^;Kat6b^E/E^* atlas appears to be thinner. (C) Representative images of dissected cervical vertebrae from C1 to C7. Note the fusion between cervical vertebrae C2 and C3, as well as C5 and C6 found in *Kat6a^E/E^;Kat6b^E/E^* fetuses (indicated as ‘fused’ in the frontal view and ‘dorsal view’). Arrow indicates ectopic bone formation in *Kat6a^E/E^* C1. aaa, anterior arch of the atlas; At, atlas; Ax, axis; C1-C7, cervical vertebrae 1-7; Eo, exoccipital bone; R1, 1st rib; rR, rudimentary rib; So, supraoccipital bone; T1, T2, thoracic vertebrae 1 and 2; TR, tympanic ring.

*Kat6a* loss-of-function embryos show a homeotic transformation resulting in eight cervical vertebrae (Voss et al., 2009). To determine the effect of point mutations on skeletal development, we examined skeletal preparations from E18.5 *Kat6a^E/E^* (*n*=5), *Kat6b^E/E^* (*n*=5), *Kat6a^E/E^;Kat6b^E/E^* (*n*=3) and WT (*n*=3) fetuses. In all genotypes the number of cervical vertebrae was normal (Fig. 4A). However, we observed abnormal development of the vertebrae in fetuses with the *Kat6a^E/E^* genotype. The *Kat6a^E/E^;Kat6b^E/E^* atlases lacked the characteristic morphology and were thin compared to the WT atlases. The *Kat6a^E/E^* atlases likewise had an abnormally slender appearance (Fig. 4B). In all *Kat6a^E/E^;Kat6b^E/E^* fetuses (*n*=3) we observed fusion between some adjacent vertebrae: in one fetus C2 and C3, as well as C5 and C6 were fused (Fig. 4C). In two *Kat6a^E/E^* fetuses we observed ectopic bone formation adjacent, but not connected to, cervical vertebrae (arrow; Fig. 4C). No other skeletal abnormalities were observed. These results show that, while no homeotic transformation is present in point mutant fetuses, development of the axial skeleton is not entirely normal.

### Point mutations of the KAT domain of KAT6A and KAT6B cause a reduction in fetal liver hematopoietic stem and progenitor cells

**Fig. 5.**
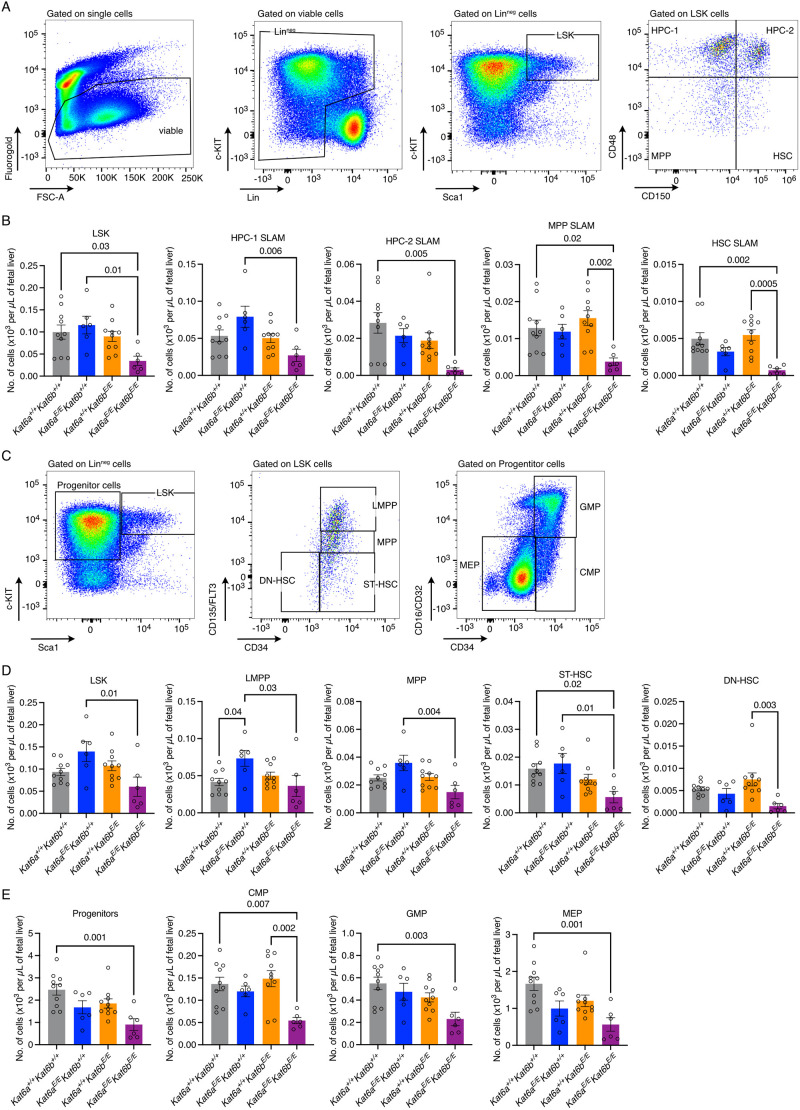
**Point mutations of the KAT domain of KAT6A and KAT6B cause a reduction in fetal liver hematopoietic stem and progenitor cells.** (A-E) Flow cytometry analysis of fetal liver hematopoietic cells from WT (*n*=10), *Kat6a^E/E^* (*n*=6), *Kat6b^E/E^* (*n*=10) and *Kat6a^E/E^;Kat6b^E/E^* (*n*=6) E14.5 fetuses. (A) Gating strategy to quantify stem and progenitor populations using SLAM markers. (B) Numbers of stem and progenitor cells analyzed using SLAM cell surface phenotype. (C) Gating strategy to quantify stem and progenitor populations using CD34, FLT3 and CD16/CD32. (D) Numbers of stem and progenitor cells analyzed using the cell surface markers in C. (E) Myeloid progenitors quantified using the cell surface markers in C. Data are presented as mean±s.e.m. Each circle represents one mouse (B-E). Data were analyzed using one-way ANOVA with Tukey's multiple comparisons test (B-E). CMP, common myeloid progenitor; DN-HSC and ST-HSCs, populations enriched in long-term repopulating and short-term repopulating hematopoietic stem cells, respectively, defined using CD34 and Flt3 markers; GMP, granulocyte-macrophage progenitor; HSC, population enriched in hematopoietic stem cells; HPC-1 and HPC-2, hematopoietic progenitor cells as defined by Kiel et al. (2005); LMPP, lymphoid primed multipotent progenitors; LSK, lineage-negative, SCA1-positive, c-KIT-positive cells; MEP, megakaryocyte-erythroid progenitor; MPP, multipotent progenitor cells; SLAM, signaling lymphocyte activation molecule family cell surface markers. Cell surface markers used to identify these populations are shown in Table S16.

We have previously shown that germline loss of KAT6A leads to the complete absence of HSCs (Thomas et al., 2006). To examine if loss of acetyltransferase activity in KAT6A, or KAT6A and KAT6B together, phenocopies a null KAT6A phenotype, we examined fetal liver hematopoiesis. We analyzed fetal liver hematopoietic cells from WT, *Kat6a^E/E^*, *Kat6b^E/E^* and *Kat6a^E/E^;Kat6b^E/E^* E14.5 fetuses. We observed a marked reduction in the number of HSCs and progenitors, as defined by their SLAM cell surface marker phenotype (Fig. 5A; Fig. S3; Table S16). *Kat6a^E/E^;Kat6b^E/E^* HSC SLAMs were reduced to 14% compared to WT samples (*P*=0.002) and to *Kat6b^E/E^* samples (*P*=0.0005), but not significantly different from *Kat6a^E/E^* samples (Fig. 5B). Interestingly, the number of HSCs in *Kat6a^E/E^* fetal livers was not statistically significantly reduced compared to WT or *Kat6b^E/E^* livers.

Using an alternative set of cell surface markers (Fig. 5C; Fig. S4) that identify stem cell activity by lack of CD34 and FLT3, we observed a reduction in the number of long-term repopulating HSCs (double negative, DN-HSCs) in *Kat6a^E/E^;Kat6b^E/E^* compared to *Kat6b^E/E^* fetal livers (*P*=0.003) but not *Kat6a^E/E^* or WT fetal livers (Fig. 5D). The number of short-term repopulating HSCs (ST-HSCs) were significantly reduced in *Kat6a^E/E^;Kat6b^E/E^* compared to *Kat6a^E/E^* or WT fetal livers (*P*=0.02 and 0.01, respectively; Fig. 5D). The number of LSK cells, lymphoid primed multipotent progenitors (LMPPs) and multipotent progenitor cells (MPPs) were reduced per liver in *Kat6a^E/E^;Kat6b^E/E^* fetuses compared to *Kat6a^E/E^* fetuses (*P*=0.01, *P*=0.03, *P*=0.004, respectively; Fig. 5D). Myeloid progenitors, common myeloid progenitors (CMP), granulocyte-macrophage progenitors (GMP) and megakaryocyte-erythroid progenitors (MEP) were significantly reduced per liver in *Kat6a^E/E^;Kat6b^E/E^* fetuses compared to wild type (*P*=0.001, *P*=0.007, *P*=0.003, *P*=0.001, respectively; Fig. 5E). Together, these results show that the stem cell compartment of fetal livers in *Kat6a^E/E^;Kat6b^E/E^* fetuses is impaired, affecting the production of progenitors of all blood cell lineages examined.

### Point mutations of the KAT domain of KAT6A and KAT6B cause a reduction in stem cell activity as assessed in competitive repopulation of the hematopoietic system

**Fig. 6.**
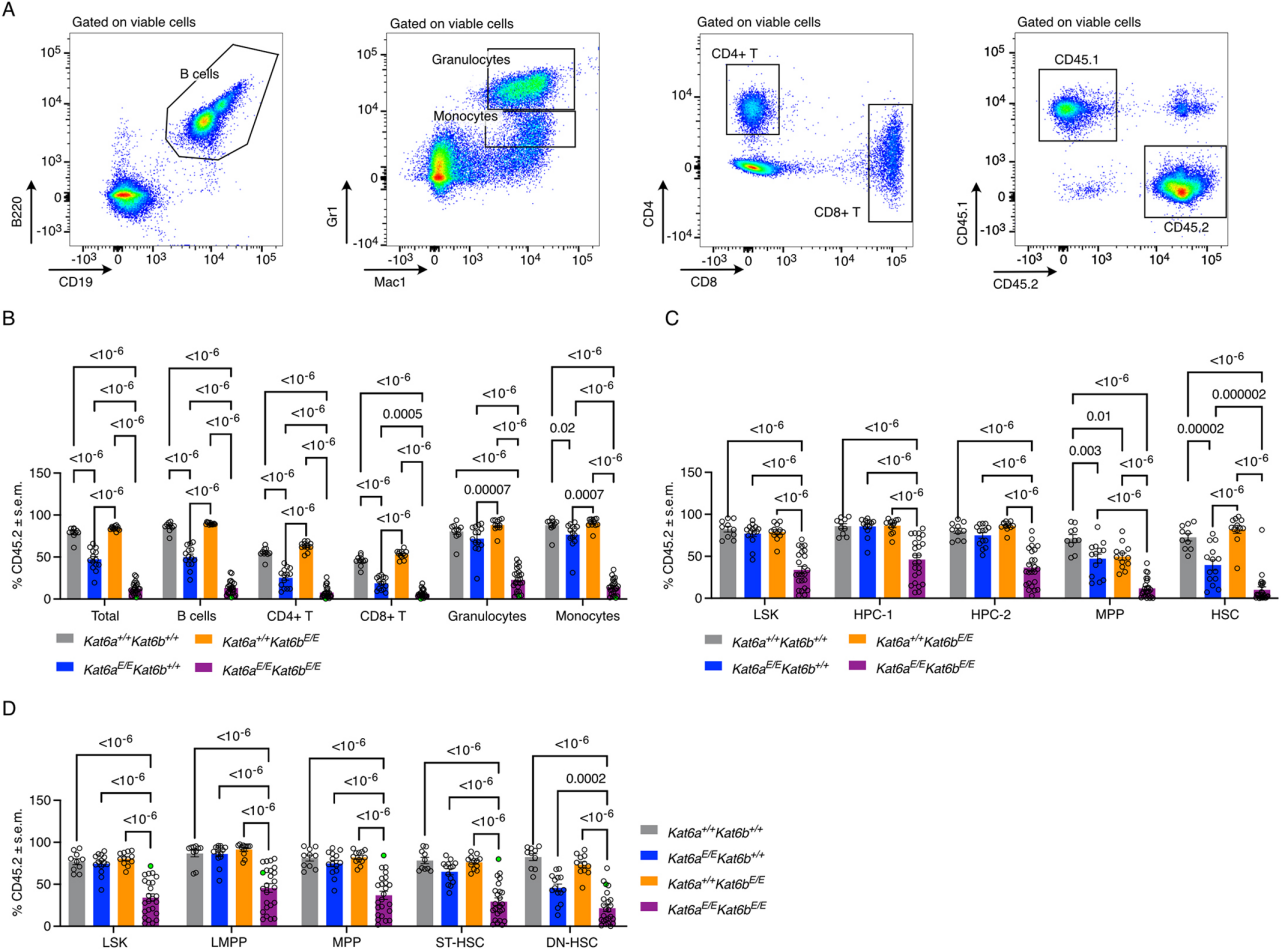
**Loss of KAT6A and KAT6B KAT activity reduces multi-lineage long-term repopulating activity in hematopoietic stem cells in primary transplants.** (A-D) Flow cytometry analysis of lethally irradiated recipient mice transplanted with E14.5 fetal liver hematopoietic cells of the following genotypes: WT (*n*=10), *Kat6a^E/E^* (*n*=14), *Kat6b^E/E^* (*n*=12) and *Kat6a^E/E^;Kat6b^E/E^* (*n*=24). (A) Gating strategy used to analyze peripheral blood of recipient mice. (B-D) Percentage of donor cell (test cell) contribution (cell surface phenotype CD45.2) to peripheral blood B, T and myeloid cells 16 weeks after transplantation (B), bone marrow stem and progenitor cells analyzed using SLAM cell surface markers, as defined by Kiel et al. (2005) (C) and bone marrow stem and progenitor cells using CD34, FLT3 and CD16/CD32 cell surface markers (D). Data are presented as mean±s.e.m. calculated from the average value of the recipients receiving fetal liver cells of the same genotype. Each circle represents a single recipient. Data were analyzed by two-way ANOVA with Tukey's multiple comparisons test (B-D). HSC, population enriched in hematopoietic stem cells; HPC-1, hematopoietic progenitor population 1; HPC-2, hematopoietic progenitor population 2; DN-HSC and ST-HSCs, populations enriched in long-term repopulating and short-term repopulating hematopoietic stem cells, respectively, defined using CD34 and Flt3 markers; LMPP, lymphoid primed multipotent progenitors; LSK, lineage-negative, SCA1-positive, c-KIT-positive cells; MPP, multipotent progenitor cells. Full gating strategy details are in Figs S5-S7.

Fetal liver cells from KAT6A loss-of-function mice do not contain hematopoietic repopulating capacity, which is reduced in heterozygous KAT6B loss-of-function fetuses. To determine if the E14.5 fetal liver cells of point mutants can repopulate the hematopoietic system of lethally irradiated mice, we performed competitive transplants in which 5×10^5^ test fetal liver cells were mixed with 10^6^ adult bone marrow competitor cells. The fetal liver cells had a CD45.2 cell surface phenotype, whereas the recipients and the competitor cells were CD45.1, allowing us to distinguish test and competitor cells (Figs S4-S8; Table S16). Each fetal liver sample was injected into three lethally irradiated recipient mice. We assessed the peripheral blood at 1 month (Fig. S4B) and at 16 weeks (Fig. 6A,B) after transplantation. Bone marrow (Fig. 6C,D; Fig. S5,S6), spleen (Fig. S7) and thymus (Fig. S8) were assessed at 16 weeks.

We found a significant reduction in both the short-term (Fig. S4B) and long-term repopulating ability of *Kat6a^E/E^;Kat6b^E/E^* double point mutant compared to WT fetal liver cells, with a reduction to 16% of WT in contribution from these cells to the peripheral blood at 16 weeks post injection (*P*<10^−6^; Fig. 6B). The reduced contribution affected all leukocyte cell types (*P*<10^−6^ for all; Fig. 6B). There was a reduction in contribution from *Kat6a^E/E^* fetal liver cells to lymphoid cells (*P*<10^−6^; Fig. 6B) and a smaller reduction in contribution to monocytes (*P*=0.02; Fig. 6B). The contribution from *Kat6b^E/E^* fetal liver cells to peripheral blood was not significantly different from WT control cells (Fig. 6B). There was a substantial reduction in contribution to the bone marrow stem cell compartment from *Kat6a^E/E^;Kat6b^E/E^* fetal liver cells (Fig. 6C). Contribution from *Kat6a^E/E^;Kat6b^E/E^* fetal liver cells to HSCs, defined by SLAM markers, was reduced to 14% compared to WT control cells (*P*<10^−6^; Fig. 6C), although one recipient had almost normal contribution. *Kat6a^E/E^;Kat6b^E/E^* fetal liver cells also showed a reduced contribution to other primitive progenitor populations, in particular an 83% reduction in contribution to MPPs (*P*<10^−6^; Fig. 6C). The contribution of *Kat6a^E/E^* fetal liver cells to the bone marrow HSC compartment was reduced, as was the contribution to MPPs (*P*=0.00002, *P*=0.003, respectively; Fig. 6C). The contribution of *Kat6b^E/E^* fetal liver cells to the bone marrow stem cell compartment of recipient mice did not significantly differ from WT control cells, except for MPPs (*P*=0.01; Fig. 6C). Similar results were obtained when we used CD34 and FLT3 to subdivide the bone marrow LSK population, although the magnitude of the reduction in contribution from *Kat6a^E/E^;Kat6b^E/E^* fetal liver cells to the CD34 and FLT3 double-negative population was less pronounced (74%; *P*<10^−6;^ Fig. 6D). Consistent with the reduction in peripheral blood myeloid cells, the reduction in contribution from *Kat6a^E/E^;Kat6b^E/E^* fetal liver cells to bone marrow CMPs, GMPs and MEPs was highly significant (all *P*<10^−6^; Fig. S5C). Congruent with the reduction in peripheral blood lymphoid cells, we observed a reduction in contribution from *Kat6a^E/E^;Kat6b^E/E^* fetal liver cells to bone marrow B-cells (all *P*<10^−6^; Fig. S6B). The contribution to the bone marrow B cell progenitors from *Kat6a^E/E^* fetal liver cells was significantly reduced compared to control cells (*P*<10^−6^ to 2×10^−5^; Fig. S6B).

As expected, given the multilineage hematopoietic defect in *Kat6a^E/E^;Kat6b^E/E^* fetal liver cells, B cell development in the spleen and T cell development in the thymus were also significantly impaired (*P*<10^−6^ to 0.001; Figs S7B, S8B,C), while *Kat6a^E/E^* fetal liver cell displayed reduced B cell development in the spleen (*P*<10^−6^ to 0.007; Fig. S7B).

### The loss of stem cell activity caused by point mutations of the KAT domain of KAT6A and KAT6B is prominent in secondary transplants assays

**Fig. 7.**
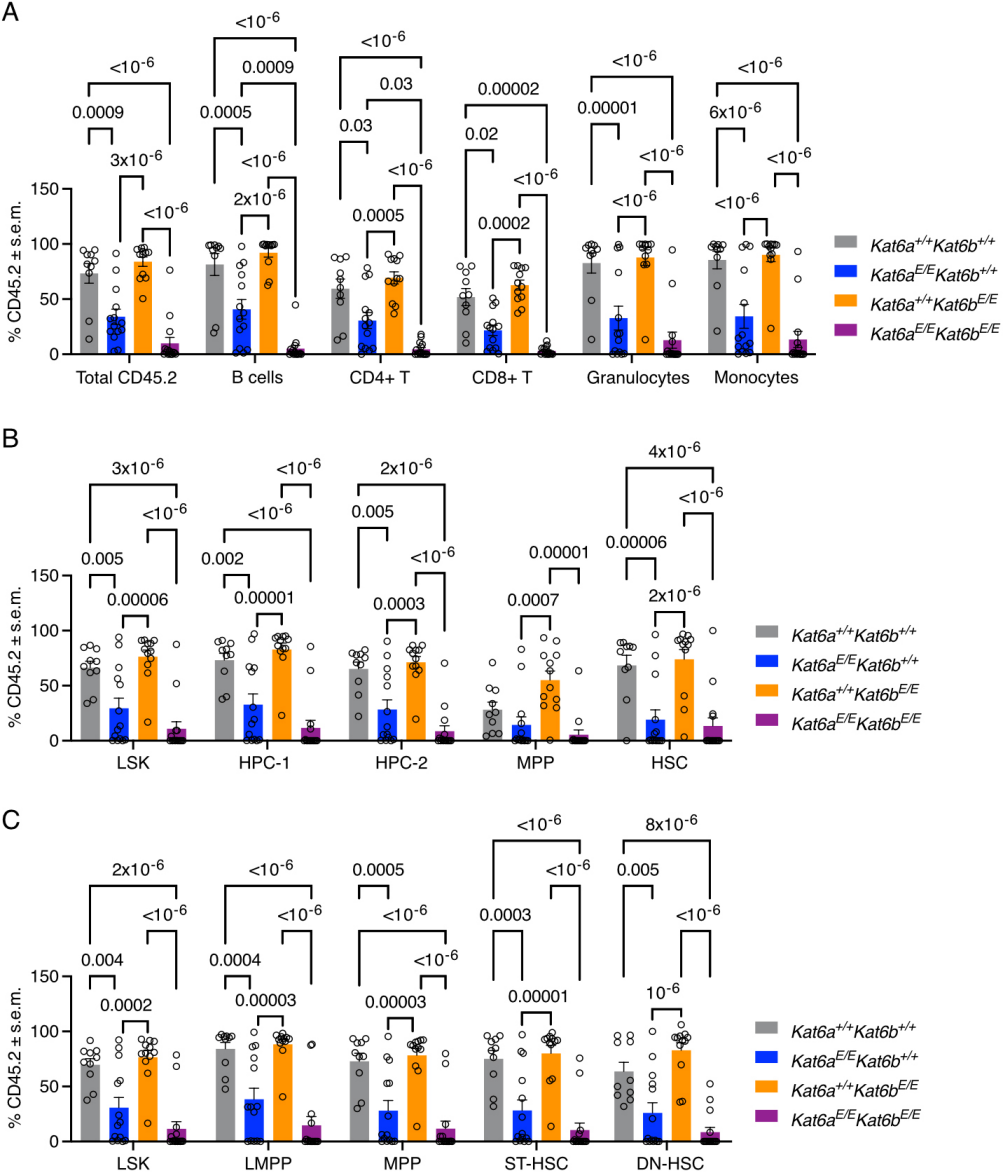
**Secondary transplants of *Kat6a;Kat6b* double point mutants show further loss of stem cell activity.** (A-C) Flow cytometry analysis of lethally irradiated secondary recipient mice transplanted with bone marrow cells from the primary recipient of fetal liver hematopoietic cells of the following genotypes: WT (*n*=10), *Kat6a^E/E^* (*n*=14) *Kat6b^E/E^* (*n*=12) and *Kat6a^E/E^;Kat6b^E/E^* (*n*=24). (A-C) Percentage of donor cell (test cell) contribution (cell surface phenotype CD45.2) to peripheral blood B, T and myeloid cells 16 weeks after transplantation (A), bone marrow stem and progenitor cells analyzed using SLAM cell surface markers, as defined by Kiel et al. (2005) (B) and bone marrow stem and progenitor cells using CD34, FLT3 and CD16/CD32 cell surface markers (C). Data are presented as mean±s.e.m. calculated from the average value of the recipients receiving fetal liver cells of the same genotype. Each circle represents a single recipient. Data were analyzed by two-way ANOVA with Tukey's multiple comparisons test (A-C). DN-HSC and ST-HSCs, populations enriched in long-term repopulating and short-term repopulating hematopoietic stem cells, respectively, defined using CD34 and Flt3 markers; HSC, population enriched in hematopoietic stem cells; HPC-1 and HPC-2, hematopoietic progenitor cells; LMPP, lymphoid primed multipotent progenitors; LSK, lineage-negative, SCA1-positive, c-KIT-positive cells; MPP, multipotent progenitor cells. Full gating strategy details in Figs S5-S7.

To apply a more stringent test of stem cell activity we undertook secondary transplant assays. Bone marrow (5×10^6^ cells) from each primary recipient that had a detectable primary transplant contribution was transplanted into one secondary recipient mouse. Peripheral blood analysis showed a marked reduction in contribution of the *Kat6a^E/E^;Kat6b^E/E^* cells, which on average was only 7.1% of WT (*P*<10^−6^; Fig. 7A; Fig. S9A). There was a large variation in contribution, with most recipients receiving no detectable contribution, two recipients displaying a modest contribution and one recipient having a level of contribution similar to WT controls. Interestingly, while two recipients had high contributions to myeloid cells, the contribution to lymphocytes was low, with only one secondary recipient displaying moderate contribution to B cells (Fig. 7A). There was reduced contribution from *Kat6a^E/E^* cells to peripheral blood in the secondary transplant recipients (*P*<0.0009; Fig. 7A), with a large variation – ranging from nearly undetectable to near WT levels of contribution (Fig. 7A). Examination of the bone marrow stem and progenitor cell compartment using SLAM markers closely reflected the peripheral blood (Fig. 7B). Similar results were obtained using CD34 and FLT3 cell surface markers to subdivide the stem and progenitor cell compartments (Fig. 7C). Examination of myeloid progenitors showed the reduction expected from the peripheral blood leucocyte profile (Fig. S9B), as did the examination of bone marrow (Fig. S9C) and spleen B cell development (Fig. S9D), as well as T cell development in the thymus (Fig. S9E).

### Mutations in the acetyltransferase domain of KAT6A cause an increase in the expression of cytokine and inflammatory response genes in fetal liver LSK cells

**Fig. 8.**
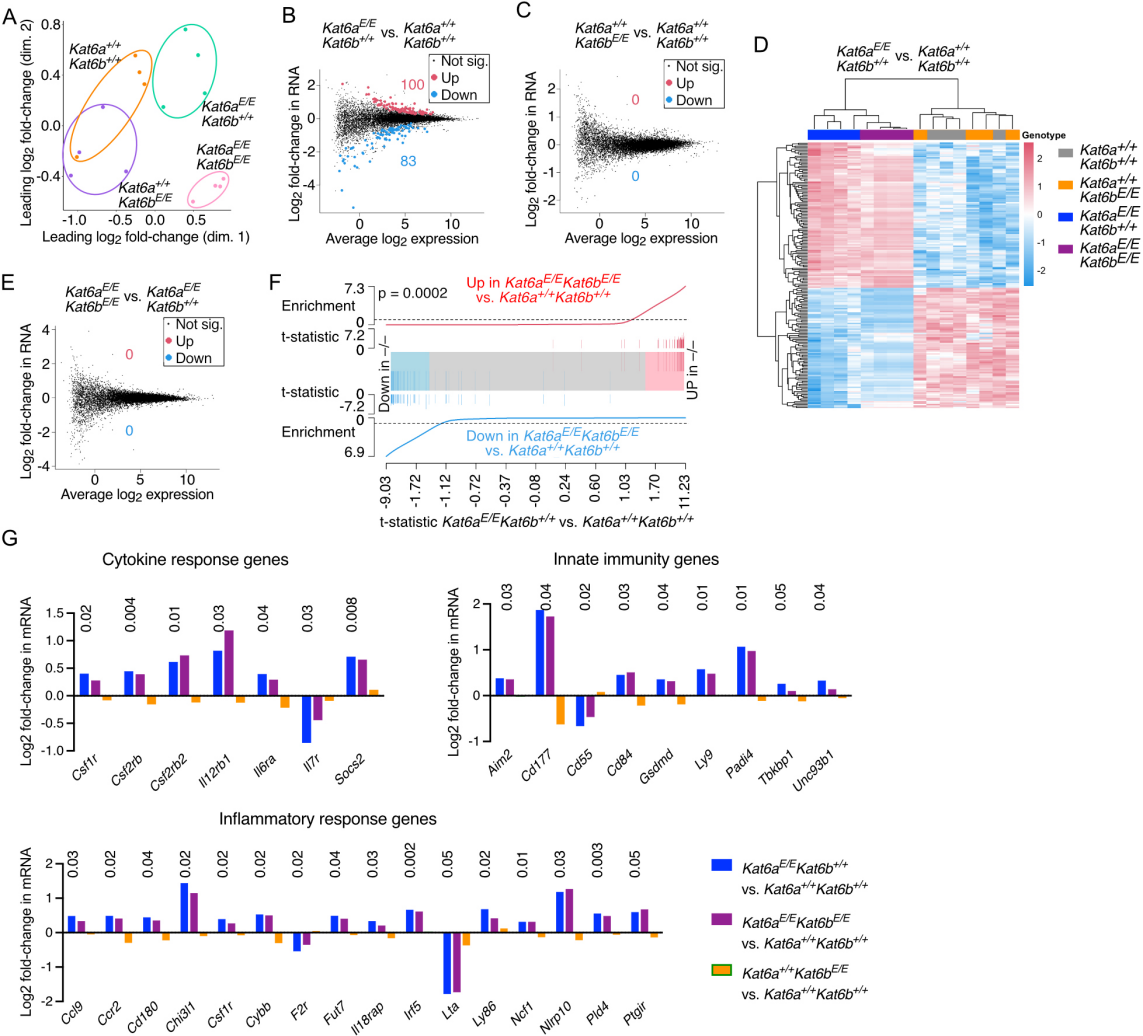
**KAT6A point mutations cause an increase in the expression of cytokine and inflammatory response genes in fetal liver LSK cells.** (A-G) LSK cells were used for RNA-sequencing. *n*=4 E14.5 fetuses per genotype. FDR<5% was considered significant. (A) Multidimensional scaling plot of the leading gene expression differences (dimension 1 and 2) between samples in pair-wise comparisons of embryos of all four genotypes. (B,C) MA plots of genes expressed in LSK cells; genotypes as indicated, genes not significantly changed indicated in black. (D) Heatmap showing genes differentially expressed in *Kat6a^E/E^* versus WT LSK cells, but also displaying results for the other genotypes. (E) MA plot of genes expressed in *Kat6a^E/E^;Kat6b^E/E^* versus *Kat6a^E/E^* LSK cells. Note absence of differentially expressed genes. (F) Barcode plot showing a positive correlation (*P*=0.0002) between gene expression changes in *Kat6a^E/E^;Kat6b^E/E^* versus WT (including genes *P*<0.01) and *Kat6a^E/E^* versus WT (including genes FDR<0.05) LSK cells. (G) Log_2_ fold-change in RNA levels of cytokine response genes, innate immunity genes and inflammatory response genes in LSK cells. FDR<0.05 shown above the bars.

To examine the effects of loss of the histone acetyltransferase activity of KAT6A or KAT6B on gene expression in hematopoietic stem and progenitor cells, we performed RNA sequencing on E14.5 fetal liver LSK cells (Tables S13-S15). Multidimensional scaling revealed the clustering, particularly of the *Kat6a^E/E^;Kat6b^E/E^* samples within genotype and segregation away from the other genotypes in dimensions 1 and 2 (Fig. 8A). *Kat6a^E/E^;Kat6b^E/E^* samples segregated from *Kat6b^E/E^* and WT samples in dimension 1 and 2 and from *Kat6a^E/E^* samples in dimension 2. We also found that 83 downregulated genes and 100 upregulated genes were differentially expressed between *Kat6a^E/E^* and WT fetal liver cells (Fig. 8B; Table S13). No genes were differentially expressed between *Kat6b^E/E^* and WT fetal liver cells (Fig. 8C; Table S14).

Unsupervised hierarchical clustering revealed that, with respect to genes differentially expressed between *Kat6a^E/E^* and WT fetal liver cells, *Kat6a^E/E^* and *Kat6a^E/E^;Kat6b^E/E^* samples were similar to each other and distinct from *Kat6b^E/E^* and WT samples (Fig. 8D). Remarkably, no genes were differentially expressed between *Kat6a^E/E^* and *Kat6a^E/E^;Kat6b^E/E^* samples (Fig. 8E). Enrichment analysis showed that the *Kat6a^E/E^* single point mutation affected gene expression in a similar way as the *Kat6a^E/E^;Kat6b^E/E^* double point mutation (Fig. 8F).

Gene Ontology analysis of the genes affected by the *Kat6a^E/E^* point mutation in the opposite direction compared with the *Kat6b^E/E^* point mutation revealed that the loss of KAT6A histone acetyltransferase activity uniquely activated genes involved in cytokine signaling, inflammatory responses and innate immunity (Fig. 8G). The specificity of the *Kat6a^E/E^* point mutation in affecting cytokine signaling, inflammatory responses and innate immunity genes suggests that the KAT6A histone acetyltransferase activity is required to restrain expression of genes typical of more differentiated cell functions.

## DISCUSSION

We set out to investigate the contribution of the lysine acetyltransferase function of KAT6A to the roles of KAT6A in embryonic and fetal development and its relationship to the acetyltransferase function of KAT6B. Our results show that a mutation in the KAT domain of KAT6A does not phenocopy the effects of a complete loss of KAT6A (Table 1). The combination of the *Kat6b^E^* point mutation with *Kat6a^E^* point mutation increased the severity of the phenotypes observed but was not as severe as the null phenotype in KAT6A, despite the high degree of redundancy in function between KAT6A and KAT6B. Two developmental processes disrupted by the complete loss of KAT6A, namely cardiac septum development and hematopoietic development, were severely affected by the KAT domain mutations in KAT6A and KAT6B (Table 1). In contrast, other developmental processes dependent on KAT6A, namely body segment identity specification and aortic arch development, were able to proceed in fetuses with mutations in the KAT6A and KAT6B KAT domains (Table 1), suggesting that lysine acetylation is not the only essential function that KAT6A and KAT6B perform.

**Table 1.** Comparison of major phenotypic anomalies between mouse mutants used in this study

Mutation	Cranio-facial abnormalities	Axial skeleton	Aortic arch	VSD	HSC	Fibroblast proliferation
*Kat6a^–/–^*	Present^1^	Homeotic transformation^2^	Interrupted type B^3^	Present^3^	Absent^4^	Fibroblast senescence^5^
*Kat6b^–/–^*	Present^6^	Normal^6^	Normal	Normal	Reduced^7^	Normal^8^
*Kat6a^E/E^*	Normal	Normal	Normal	Present	Reduced	Fibroblast senescence^9^
*Kat6b^E/E^*	Normal	Normal	Normal	Normal	Normal	N/E
*Kat6a^E/E^; Kat6b^E/E^*	Normal	Normal	Normal	Present	Severely reduced	Fibroblast senescence

^1^Vanyai et al., 2019; ^2^Voss et al., 2009; ^3^Voss et al., 2012; ^4^Thomas et al., 2006; ^5^Sheikh et al., 2015c; ^6^Thomas et al., 2000; ^7^Bergamasco et al., 2024a; ^8^Bergamasco et al., 2025a; ^9^Perez-Campo et al., 2014. HSC, hematopoietic stem cells; N/E not examined; VSD, ventricular septal defects.

The effect of the *Kat6a* point mutation on H3K23ac levels was indistinguishable from the null mutation. It is noteworthy that low level of non-enzymatic acetylation of proteins by acetyl-CoA can occur (Olia et al., 2015). As expected, since KAT6 inhibitors induce senescence (Baell et al., 2018), fibroblasts isolated from KAT6A point mutant embryos became senescent even when grown in 3% O_2_ – conditions that allow mouse embryonic fibroblasts to grow indefinitely in culture (Parrinello et al., 2003). From this we conclude that the point mutation reduces acetylation to background levels.

*Kat6a* null embryos fail to develop definitive HSCs (Katsumoto et al., 2006; Sheikh et al., 2016; Thomas et al., 2006). We found a reduction in transplantable HSC activity in KAT6A point mutant fetuses, reflecting a reduction in fetal cells with an HSC cell surface phenotype, as previously reported (Perez-Campo et al., 2009). Our results in KAT6A point mutant fetuses show a somewhat less severe phenotype than reported by Perez-Campo et al. (2009); this likely is due to differences in how the competitive transplantation experiments were performed. In our hands the point mutation produced a similar effect to a loss of one allele of *Kat6a*, namely about a 50% reduction in transplantation activity compared to WT mice. (Thomas et al., 2006). Secondary transplants of KAT6A point mutant cells led to a further reduction in contribution to recipients. KAT6B is also necessary for normal HSC activity (Bergamasco et al., 2024a) and the KAT6A and KAT6B double point mutation led to a further reduction in competitive transplant activity. Interestingly, in secondary transplants of double point mutant cells, while cells from most donors entirely failed to contribute to the recipient, in two cases there was significant contribution, actually more than in the primary transplants, suggesting that the WT bone environment of the primary recipient mice had ameliorated the stem cell defects.

The homeotic transformation of the axial skeleton found in KAT6A null mutants is not present in *Kat6a^E/E^* or *Kat6a^E/E^;Kat6b^E/E^* double point mutants. In embryos lacking KAT6A completely, we have shown a reduction in Hox gene expression, a posterior shift in Hox gene expression and a homeotic transformation affecting 19 body segments (Voss et al., 2009). In *Kat6a^E/E^* or *Kat6a^E/E^;Kat6b^E/E^* point mutant embryos, the gene expression changes are less pronounced, although highly correlated to the gene expression changes in *Kat6a^–/–^* embryos. This suggests that any change in Hox gene expression does not reach the threshold required to alter specification of axial segments, even in the absence of histone acetylation activity in both KAT6A and KAT6B together. This is evidence that gene regulatory activity is retained by the KAT6A-containing complex lacking enzymatic activity.

Heterozygous loss of KMT2A (MLL1) causes an anterior homeotic transformation of cervical vertebrae and a posterior shift in cervical Hox gene expression (Yu et al., 1995). We previously observed a reduction in KMT2A recruitment to Hox loci in the absence of KAT6A (Voss et al., 2009). Recently, we have shown reduced KMT2A recruitment to target loci, in particular homeodomain proteins, in neural stem cells in the absence of KAT6A (Voss et al., 2026). Like KAT6A, KMT2A has crucial roles in HSCs (McMahon et al., 2007; Yu et al., 1995). In cord blood cells it has been reported that KAT6A can be recruited to Hox loci through an association with methylated H3K4, which is generated by KMT2A, and that depletion of KMT2A results in loss of KAT6A at these loci (Paggetti et al., 2010). Disruption of the interaction between KMT2A- and KAT6A-mediated histone modifications has been implicated in the pathogenesis of cancer (Hemming et al., 2022; Miyamoto et al., 2020; Olsen et al., 2025; Sheridan et al., 2024) and it is interesting to note that tissue-specific lack of KMT2D results in a VSD (Ang et al., 2016). These results point to an emerging model in which a series of chromatin modifications mediated by KAT6A and KMT2A complexes acting in concert prepare a locus for transcription (Yokoyama, 2022). In the light of this model, our results suggest that, in some developmental processes, the presence of an intact complex, containing a KAT6A without acetyltransferase activity but with the necessary chromatin binding domains, can still allow key events in the organization of chromatin required for embryogenesis to proceed. In the case of the axial skeleton, this suggests that KMT2A is recruited to Hox loci with the appropriate timing necessary for coordinated expression of Hox genes with the appropriate anterior expression boundaries. In contrast, in the HSC compartment, the loss of KAT activity from both KAT6A and KAT6B almost eliminates definitive HSC activity.

We have previously shown that complete loss of KAT6A leads to a DiGeorge syndrome-like anomalies, including an interrupted aortic arch type B, VSDs, cranio-facial defects, cleft palate and hypoplastic thymus (Thomas et al., 2006; Vanyai et al., 2019, 2015; Voss et al., 2012). Loss of TBX1 function is necessary and sufficient for the development of DiGeorge syndrome (Lindsay et al., 2001). We showed that *Tbx1* is a target gene of KAT6A. *Kat6a^E/E^* and *Kat6a^E/E^;Kat6b^E/E^* pups did not display DiGeorge syndrome-like anomalies, except for the presence of VSDs. RNA-sequencing showed a reduction of *Tbx1* gene expression in *Kat6a^–/–^* null mutants at E9.5 as previously reported (Voss et al., 2012). In *Kat6a^E/E^* and *Kat6a^E/E^;Kat6b^E/E^* embryos, the reduction in *Tbx1* expression and other regulators of heart development was less than in *Kat6a^–/–^* null embryos, but nevertheless below the threshold of *Tbx1* expression required for septal development. *Tbx1* is required in a dose-dependent fashion for normal heart development (Lindsay et al., 2001). Interestingly, loss of ING5 also leads to VSDs but appears to be redundant with ING4 in other aspects of KAT6A functions (Mah et al., 2024) This suggests that a complex containing ING5 and KAT6A acetyltransferase activity is necessary for sufficient *Tbx1* gene expression during ventricular septum development, but that the acetyltransferase activity is not essential in other aspects of heart and aortic arch development.

In conclusion, our results examining organs and cell types most affected by complete loss of KAT6A function show that the KAT activity is crucial for survival but does not constitute the entire sum of the functional activity of the proteins , as revealed by the *Kat6a* null phenotype. Taken together, our results suggest that, while some chromatin proteins can usefully be characterized as ‘writers’ and ‘readers’, other large, multidomain chromatin proteins may have essential roles as ‘readers’ as well as ‘writers’, potentially operating independent of one another in some cellular processes. Nevertheless, drugs targeting the KAT domain have potent anticancer activity (Mukohara et al., 2024; Sharma et al., 2023). Since some cancer cells appear to be dependent on acetyltransferase activity, this suggests that the potential side effects of drugs targeting the acetyltransferase activity may be less than might be expected from depleting the entire protein and destabilizing the KAT6 complex.

## MATERIALS AND METHODS

### Ethics

Mouse husbandry and experiments were performed in accordance with the Australian code for the care and use of animals for scientific purposes and with the approval of the Walter and Eliza Hall Animal Ethics Committee.

### Mice

Mice heterozygous for a glycine to glutamic acid mutation at position 656 of KAT6A (homologous to position 657 in human KAT6A) and at position 577 of KAT6B (isoform 1) were generated on a C57BL/6J background, using previously described methods for Cas9 directed mutagenesis (Kueh et al., 2017). A mixture of 20 ng/μl Cas9 mRNA, 10 ng/μl single guide RNA and 40 ng/μl donor template were injected into fertilized one-cell-stage C57BL/6J embryos. This resulted in mutation of the sequence GGC, coding for glycine at position 656 of KAT6A (NCBI reference sequence: NP_001074618.1) to GAA, coding for glutamic acid, causing the amino acid sequence of the acetyl-CoA binding domain, VSCIMILPQYQRK**G**YGRFLI to be converted to VSCIMILPQYQRK**E**YGRFLI, resulting in loss of acetyltransferase activity (Takechi and Nakayama, 1999). For KAT6B, the CRISPR procedure led to the mutation of the sequence GGA, coding for glycine position 577 of KAT6B (NCBI reference sequence: NP_001192170.1) to GAG, likewise coding for glutamic acid. This resulted in the amino acid sequence of the acetyl-CoA binding domain, VSCIMIMPQHQRQ**G**FGRFLI being converted to VSCIMIMPQHQRQ**E**FGRFLI. Point mutant knock-in mice, which are potentially mosaic, were backcrossed to C57BL/6J mice to generate founders, that were then backcrossed for at least five generations to eliminate potential off-target events. Mice were maintained on a C57BL/6J genetic background. Transmission of the mutant alleles was confirmed by sequencing, after which mice were genotyped by two separate PCR reactions to detect either the WT allele or the point mutant allele (Table S1). Since the mutant alleles have only a two-nucleotide substitution, the PCR band sizes are the same for the mutant and WT alleles. To detect the difference in sequence, the final two nucleotides of one of the primer pairs contain the mutant bases and so does not bind to the WT sequence and so Taq polymerase cannot amplify the WT allele (and vice versa). The *Kat6a* band is 329 bp and the *Kat6b* band is 228 bp (Fig. 1B).

The *Kat6a* null allele, *Kat6a^–^* lacking exons 3 to 7 (Voss et al., 2009), and *Kat6b* null allele, *Kat6b^–^* lacking exons 2 to 12 (Bergamasco et al., 2024a), have been previously reported. These mouse strains were maintained on a C57BL/6 background. The *Kat6b^gt/^* allele (*Querkopf*), which was generated by gene trap, produces 10% normal *Kat6b* mRNA (Thomas et al., 2000). This allele was backcrossed to C57BL/6.

Mice were fed *ad libitum* and kept in a 14-h light/10-h dark cycle at 22°C. Mice were mated and examined for vaginal plugs the following morning, with noon following the observation of a vaginal plug termed E0.5. Embryos and fetuses were collected at the developmental stages indicated in text and figure legends.

### Western blot analysis

Histones were acid extracted using 0.2 M H_2_SO_4_, collected by centrifugation (10,000 ***g***) and then dialyzed (Spectra/Por Dialysis Membrane Tubing; molecular weight cut-off 20 kDa) against 0.1 M acetic acid for 1 h at 4°C followed by ultra-pure water overnight. Acid-extracted histones were separated by gel electrophoresis on 4-12% Bis-Tris gels (Thermo Fisher Scientific, NP0322), then transferred to nitrocellulose membranes (Licor, 926-31090). Membranes were blocked for 1 h at room temperature using blocking buffer [Intercept^®^ (PBS); LICORbio, 927-70001] and probed with antibodies against H3K9ac (Epicypher, 13-0033; dilution 1:5000), H3K14ac (Abcam, ab52946; 1:1000) or H3K23ac (Millipore, 07-355; 1:5000) and pan H3 (Abcam, 10799; 1:5000) overnight at 4°C. Membranes were washed in PBS+0.1% Tween-20 (Sigma-Aldrich, P1379) and incubated with goat anti-mouse IgG (IRDye^®^ 800 CW; LICORbio, 926-32210; 1:10,000) and goat anti-rabbit IgG (IRDye^®^; LICORbio, 926-68071; 1:10,000) secondary antibodies for 1 h. Samples were imaged and analyzed using automated imager software (Odyssey Imager; LICORbio).

### Mouse embryonic fibroblast culture

Fibroblasts isolated from E12.5 embryos were cultured in Dulbecco's modified Eagle medium (Gibco, 11995) supplemented with 100 U/ml penicillin/streptomycin (Gibco, 15140122) and 10% fetal calf serum. Cells were cultured in 3% O_2_/5% CO_2_, because mouse embryonic fibroblasts can grow indefinitely without immortalization procedures in 3% O_2_ (Parrinello et al., 2003). Cell counts were determined at each passage using a Countess™ cell counter (Thermo Fisher Scientific).

### Histological and morphological analysis

Skeletal preparations of fetuses at E18.5 were processed and stained with Alizarin Red for bone and Alcian Blue for cartilage components (Thomas et al., 2000). Tissues were formalin-fixed, paraffin embedded and stained with Hematoxylin and Eosin (H&E) using standard techniques. Slides were scanned using an Olympus VS200 scanner and visualized using Path (version 5.1). External appearance and viability (heartbeat) were examined at dissection at E9.5, E14.5, E18.5 and E19.5. Cranio-facial, heart and aortic arch morphology were examined at dissection at E18.5 and E19.5.

### Hematological transplantation assays

Transplantation assays were performed as described previously (Bergamasco et al., 2024a). Briefly, for primary competitive transplantation assays, 10^6^ WT bone marrow competitor cells from congenic C57Bl/6 mice carrying the CD45.1 cell surface marker, were mixed with 5×10^5^ E14.5 fetal liver cells from test mice, which carry the CD45.2 cell surface marker; this allows competitor and test cells to be distinguished by flow cytometry. Cell number was determined using an automated hematology analyzer (Advia 2120i, Siemens Healthineers). The appropriate number of cells were mixed and suspended in a balanced salt solution supplemented with 2% fetal calf serum, then intravenously injected into lethally irradiated (two doses of 5.5 Gy separated by 3 h) C57BL/6 recipients that express the CD45.1 cell surface marker. Each sample was injected into three recipient mice in primary transplants. For secondary transplants, bone marrow from primary transplant recipients was flushed, counted and suspended in a balanced salt solution, as above, and intravenously injected into a single lethally irradiated CD45.1, C57BL/6 secondary recipient.

### Flow cytometry analysis

Cells were isolated from the fetal livers (passed through a 40 μm sieve), bone marrow, spleen, thymus and peripheral blood and suspended in a balanced salt solution supplemented with 2% fetal calf serum. Cells were then stained using antibodies against cell surface markers specific for distinct populations. These were selected from the following: B220 (clone RA3-6B2), CD4 (clone GK1.5-7), CD8a (clone 53.6.7), CD19 (clone 1D3), Gr1 (clone RB6-8C5), Mac1 (CD11b, clone M1/70), CD16/32 (clone 24G2-16), CD21 (clone 7G6), CD23 (clone B3B4), CD25 (clone PC61/F7), CD27 (clone A7), CD34 (clone RAM34), CD44 (clone IM7.8), CD45.1 (clone A20), CD45.2 (clone S450-15-2), CD48 (clone HM48-1), c-KIT (CD117, clone 2B8), Flt3 (CD135. clone A2F10-1), CD150 (SLAMF1, clone TC15-12F12.2), IgD (clone 11-26c), IgM (clone 5.1), Sca1 (Ly6A/E, clone E13,), Ter119 (clone Ly-76). Antibodies were directly conjugated to fluorophores selected for their compatibility in multicolor flow cytometric analysis – these included AlexaFluor 700, phycoerythrin (PE), fluorescein isothiocyanate (FITC), peridinin chlorophyll A (PerCP), phycoeryhtrin-cyanine dye (PeCy7), allophycocyanin (APC), AlexaFluor 647, AlexaFluor 594 or biotin. Streptavidin-conjugated secondary antibodies labelled with A700, PerCP, BV650 and PeCy7 were used for biotin-conjugated primary antibodies. Antibodies were generated in house by the Walter and Eliza Hall Institute monoclonal facility, or purchased from BD Pharmingen, eBioscience, BioLegend or Invitrogen. For exclusion of lineage-positive cells, antibodies against B220, CD3, CD4, CD8, CD19, Gr1 and Ter-119 were used. Cells were analyzed on the LSRII (BD) cytometer.

### RNA isolation and RNA-sequencing

Whole E9.5 embryos were dissected, photographed and stored in 200 μl Zymo DNA/RNA shield solution (lysed) to await genotyping using the dissected extra-embryonic membranes. Embryos of the desired genotypes were then selected based on similar external appearance (Fig. S2). E14.5 fetal livers were isolated, passed through a sieve (40 μm) and fetal liver cells were genotyped. Selected embryos and fetal liver cell isolates were subjected to total RNA extraction using an RNeasy Mini Kit (Qiagen) and eluted with 30 μl RNAse-free water. RNA yield and integrity were assessed using an automated gel electrophoresis system (Tape Station, Agilent Technologies). Then 500 ng of RNA were used to generate RNA-sequencing libraries using a library construction kit (TruSeq RNA Library Prep Kit v2, Illumina) and sequenced on a NextSeq2000 sequencer (Illumina) to give 66 bp paired end reads.

### RNA-sequencing data analysis

FastQ files were run through FastQC and MultiQC for quality assessment. Samples were aligned to the mm39 build of the mouse genome using Rsubread align (Liao et al., 2019), achieving an average mapping proportion of 98%. Rsubread featureCounts was used to count read-pairs mapping to RefSeq genes, using strict RefSeq mm39 annotation dated 11 April 2023 downloaded from https://bioinf.wehi.edu.au/Rsubread/annot/. Pseudoautosomal genes on the Y chromosome were masked to avoid duplication with the same sequences on the X chromosome. An average of 18.9 million read-pairs were uniquely assigned to genes per sample. Gene information was obtained from the ‘Mus_musculus.gene_info’ file downloaded from the NCBI on 27 June 2024. Downstream analysis was restricted to protein-coding genes and Y-chromosome genes were removed to avoid sex effects. The E9.5 and E14.5 fetal liver samples were analyzed separately. Low expressed genes were filtered using the edgeR filterByExpr function (Chen et al., 2025), resulting in 13,964 genes for the E9.5 samples and 12,853 genes for the E14.5 samples. TMM library size normalization was applied.

Differential expression analyses used limma and voomLmFit (Baldoni et al., 2024; Law et al., 2014; Ritchie et al., 2015). Four surrogate variables were estimated by the limma wsva function to adjust for unknown causes of variability. Linear models were fitted to the data using voomLmFit, with mouse litter as a random block. Robust empirical Bayes-moderated *t*-statistics were used to identify differentially expressed genes between experimental groups (Phipson et al., 2016). Genes with false discovery rate (FDR)<5% were considered to be differentially expressed. Correlations between expression profiles were displayed using the limma barcodeplot function and assessed with roast gene tests (Wu et al., 2010).

### Statistics

The methods for statistical analyses, tests and sample sizes are described in the figure legends. RNA-sequencing data were analyzed as described in the ‘RNA-sequencing data analysis’ section. Other data were analyzed using GraphPad Prism Version 10.4.1 (532), except Fisher's exact tests and two-way ANOVA, which were performed on Stata/SE 17.0, or by computing the cumulative binomial probability of being less than or equal to the expected value (pbinom) using R version 4.5.0 (2025-04-11).

## Supplementary Material

10.1242/develop.205559_sup1 Supplementary information

Table S2. Genes differentially expressed in Kat6aG656E/G656EKat6bG577E/G577E vs. Kat6a^+/+^Kat6b^+/+^ E9.5 embryos

Table S3. Genes differentially expressed in Kat6a^-/-^Kat6b^+/+^ vs. Kat6a^+/+^Kat6b^+/+^ E9.5 embryos

Table S4. Genes differentially expressed in Kat6a^+/+^Kat6b^-/-^ vs. Kat6a^+/+^Kat6b^+/+^ E9.5 embryos

Table S5. Genes differentially expressed in Kat6aG656E/G656EKat6b^+/+^ vs. Kat6a^+/+^ Kat6b^+/+^ E9.5 embryos

Table S6. Genes differentially expressed in Kat6a^+/+^Kat6bG577E/G577E vs. Kat6a^+/+^ Kat6b^+/+^ E9.5 embryos

Table S7. GO terms BP (biological process) of genes differentially expressed in Kat6a^-/-^Kat6b^+/+^ vs. Kat6a^+/+^Kat6b^+/+^ and in Kat6aG656E/G656EKat6bG577E/G577E vs. Kat6a^+/+^Kat6b^+/+^ E9.5 embryos

Table S8. GO terms BP (biological process) of genes differentially expressed in Kat6a^-/-^Kat6b^+/+^ vs. Kat6a^+/+^Kat6b^+/+^ but not in Kat6aG656E/G656EKat6bG577E/G577E vs. Kat6a^+/+^Kat6b^+/+^ E9.5 embryos

Table S9. GO terms BP (biological process) of genes differentially expressed in Kat6aG656E/G656EKat6bG577E/G577E vs. Kat6a^+/+^Kat6b^+/+^ but not in in Kat6a^-/-^Kat6b^+/+^ vs. Kat6a^+/+^Kat6b^+/+^ E9.5 embryos

Table S10. GO terms BP (biological process) of genes differentially expressed in Kat6a^+/+^Kat6b^-/-^ vs. Kat6a^+/+^Kat6b^+/+^ and in Kat6aG656E/G656EKat6bG577E/G577E vs. Kat6a^+/+^Kat6b^+/+^ E9.5 embryos

Table S11. GO terms BP (biological process) of genes differentially expressed in Kat6a^+/+^Kat6b^-/-^ vs. Kat6a^+/+^Kat6b^+/+^ but not in Kat6aG656E/G656EKat6bG577E/G577E vs. Kat6a^+/+^Kat6b^+/+^ E9.5 embryos

Table S12. GO terms BP (biological process) of genes differentially expressed in Kat6aG656E/G656EKat6bG577E/G577E vs. Kat6a^+/+^Kat6b^+/+^ but not in Kat6a^+/+^Kat6b^-/-^ vs. Kat6a^+/+^Kat6b^+/+^ E9.5 embryos

Table S13. Genes differentially expressed in Kat6aG656E/G656EKat6b^+/+^ vs. Kat6a^+/+^Kat6b^+/+^ E14.5 foetal liver haematopoietic cells

Table S14. Genes differentially expressed in Kat6a+/+Kat6bG577E/G577E vs. Kat6a^+/+^Kat6b^+/+^ E14.5 foetal liver haematopoietic cells

Table S15. Genes differentially expressed in Kat6aG656E/G656EKat6bG577E/G577E vs. Kat6a^+/+^Kat6b^+/+^ E14.5 foetal liver haematopoietic cells
