# Supplementary material for: The roles of the acetyltransferase domains of the chromatin regulators KAT6A and KAT6B in vivo
Source: Development. 2026 Jun 18;153(12):dev205559. doi: 10.1242/dev.205559 (PMC13327545; doi:10.1242/dev.205559)
Supplement: Supplementary information [file develop-153-205559-s1.pdf]

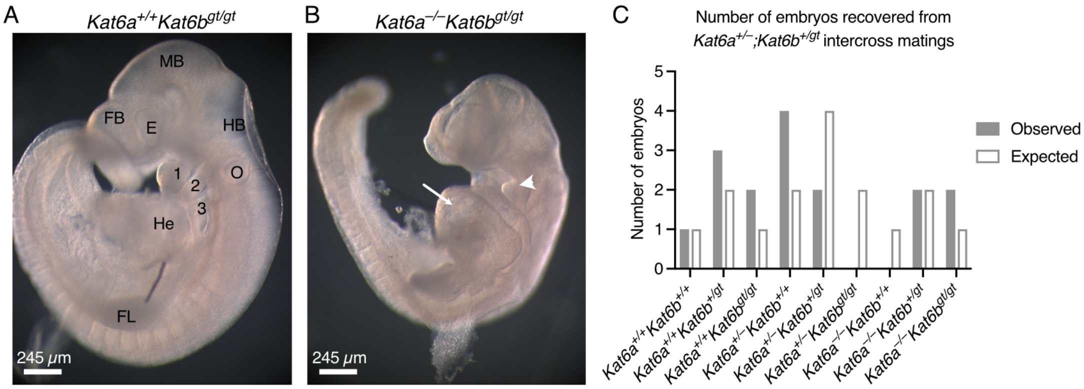

Fig. S1. External appearance and enumeration of embryos isolated at E9.5 from *Kat6a*$^{+/–}$;*Kat6b*$^{+/gt}$ x *Kat6a*$^{+/–}$;*Kat6b*$^{+/gt}$ matings. *Kat6a*$^{+/–}$; *Kat6b*$^{+/–}$ compound double heterozygous mice on a C57BL/6 genetic background are perinatal lethal (Bergamasco et al., 2024). In contrast, *Kat6a*$^{+/–}$;*Kat6b*$^{+/gt}$ survive to adulthood and can be bred, as the *Kat6b*$^{gt/}$ allele produces approximately 10% normal *Kat6a* mRNA (Thomas et al., 2000).

(A,B) Representative external appearance of a *Kat6a*$^{+/+}$;*Kat6b*$^{gt/gt}$ embryo (A) and a *Kat6a*$^{–/–}$;*Kat6b*$^{gt/gt}$ embryo. *Kat6a*$^{–/–}$;*Kat6b*$^{gt/gt}$ embryos arrested at E9.0 and displayed hypoplastic pharyngeal arches, an enlarged heart and abnormal small forebrain, midbrain and hindbrain structures and a small otic vesicle (B cf. A). Embryos of other genotypes recovered had grown to a normal size for E9.5 embryos and did not display external anomalies.

(C) Number and genotypes of embryos observed and expected. N = 16 embryos recovered.

1, 2, 3, mandibular portion of the 1st pharyngeal arch, 2nd and 3rd pharyngeal arch; E, eye; FB, forebrain; FL, forelimb; HB, hindbrain; MB, midbrain; O, otic vesicle. Arrow in (B) indicates the enlarged heart and arrowhead the hypoplastic mandibular portion of the 1st pharyngeal arch. Scale bar = 245 μm .

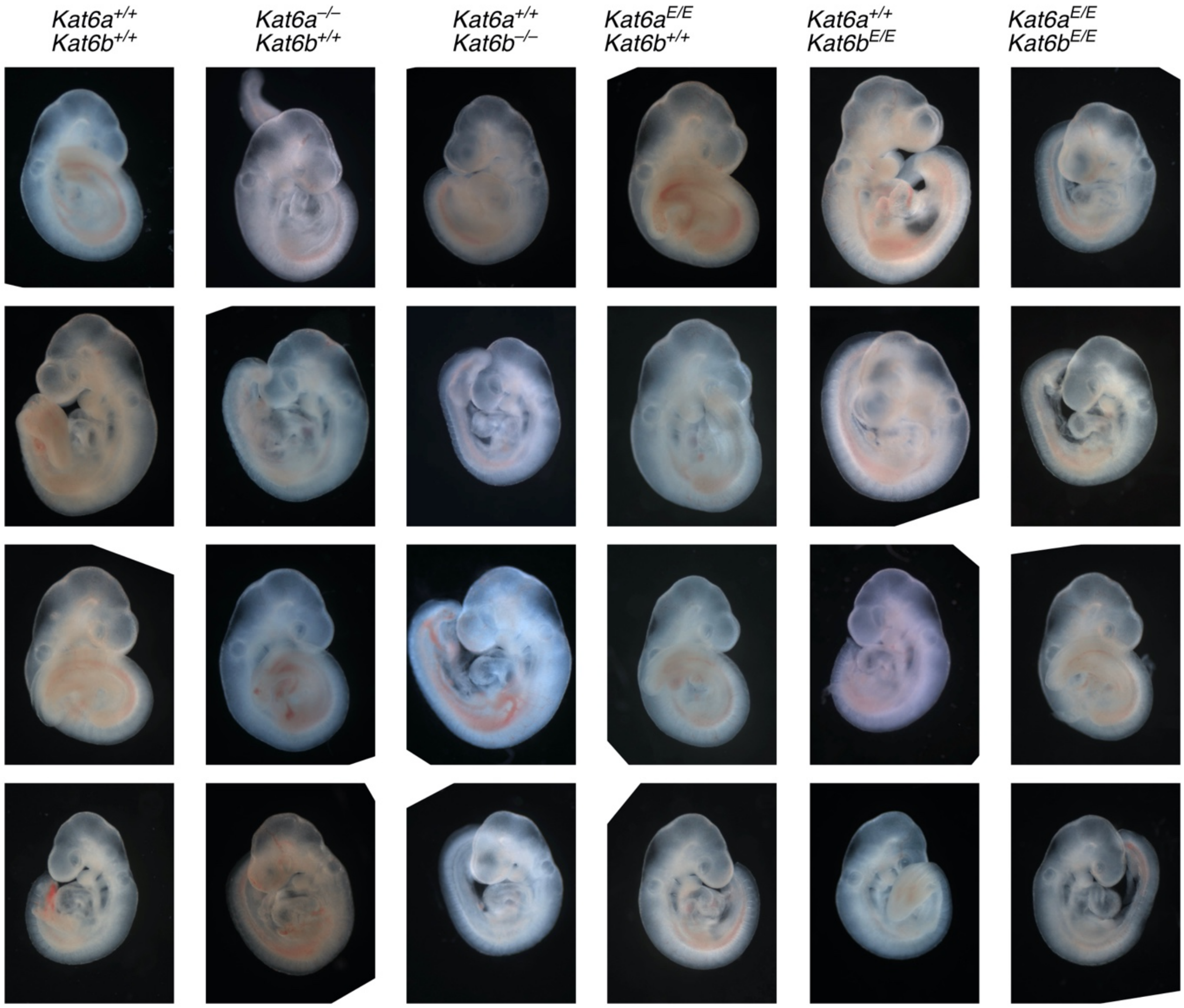

Fig. S2. External appearance of embryos used for RNA sequencing. *Kat6a*$^{+/+}$;*Kat6b*$^{+/+}$, *Kat6a*$^{-/-}$;*Kat6b*$^{+/+}$, *Kat6a*$^{+/+}$;*Kat6b*$^{-/-}$, *Kat6a*$^{E/E}$;*Kat6b*$^{+/+}$, *Kat6a*$^{+/+}$;*Kat6b*$^{E/E}$, and *Kat6a*$^{E/E}$;*Kat6b*$^{E/E}$ E9.5 embryos were used for RNA sequencing. Genotypes as indicated. N = 4 E9.5 embryos per genotype were used. All embryos were male.

Development • Supplementary information

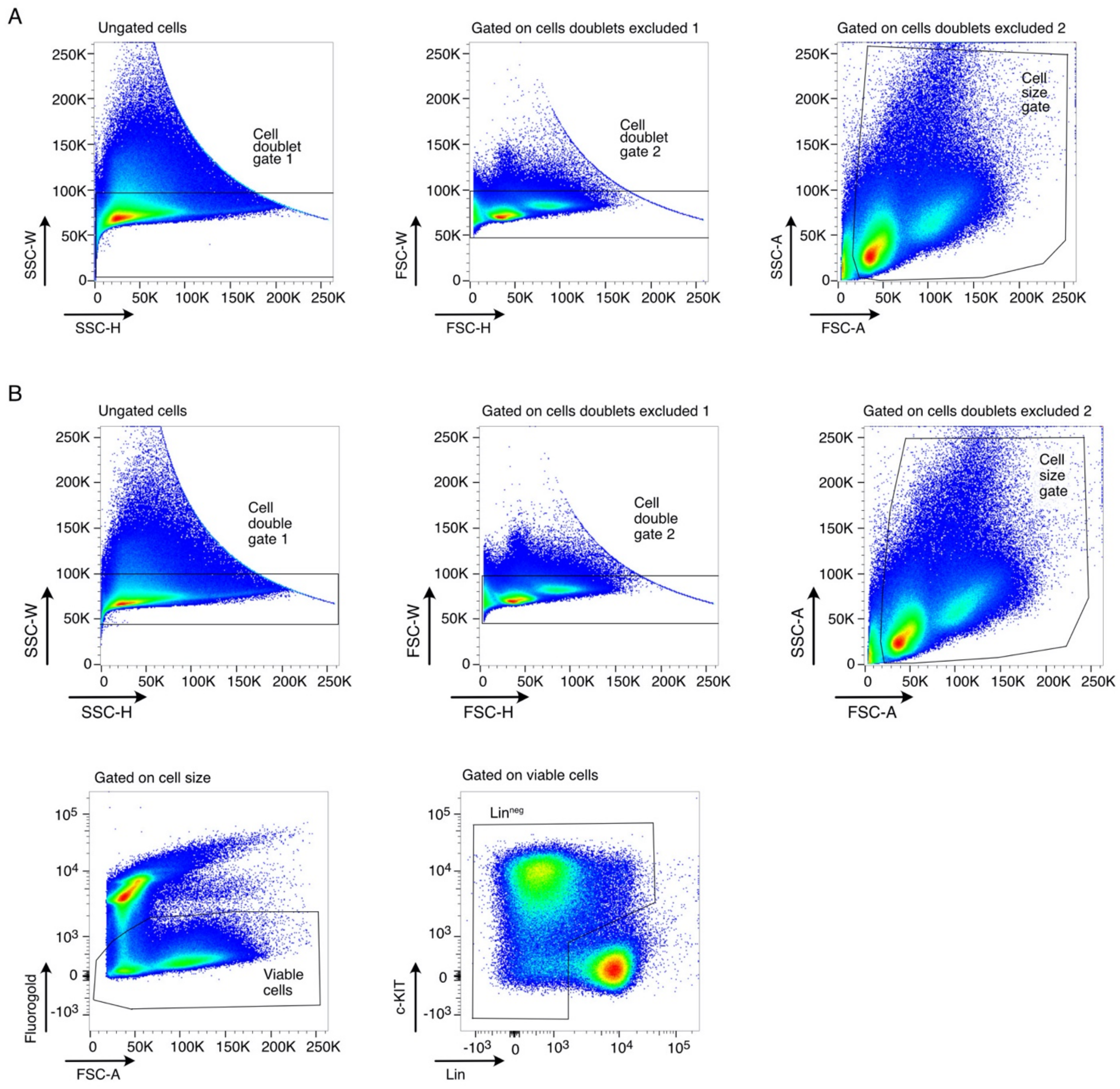

Fig. S3. Gating strategy for the flow cytometric analysis of fetal liver haematopoietic cells. (A) Cell doublet exclusion and cell size gates prior to gating as shown in Figure 5A for SLAM markers.

(B) Cell doublet exclusion, cell size, cell viability and c-KIT positive and lineage marker negative gates prior to gating as shown in Figure 5C for stem and progenitor cells based on CD135/FLT3 and CD34, as well as CD16/CD32 and CD34.

Development • Supplementary information

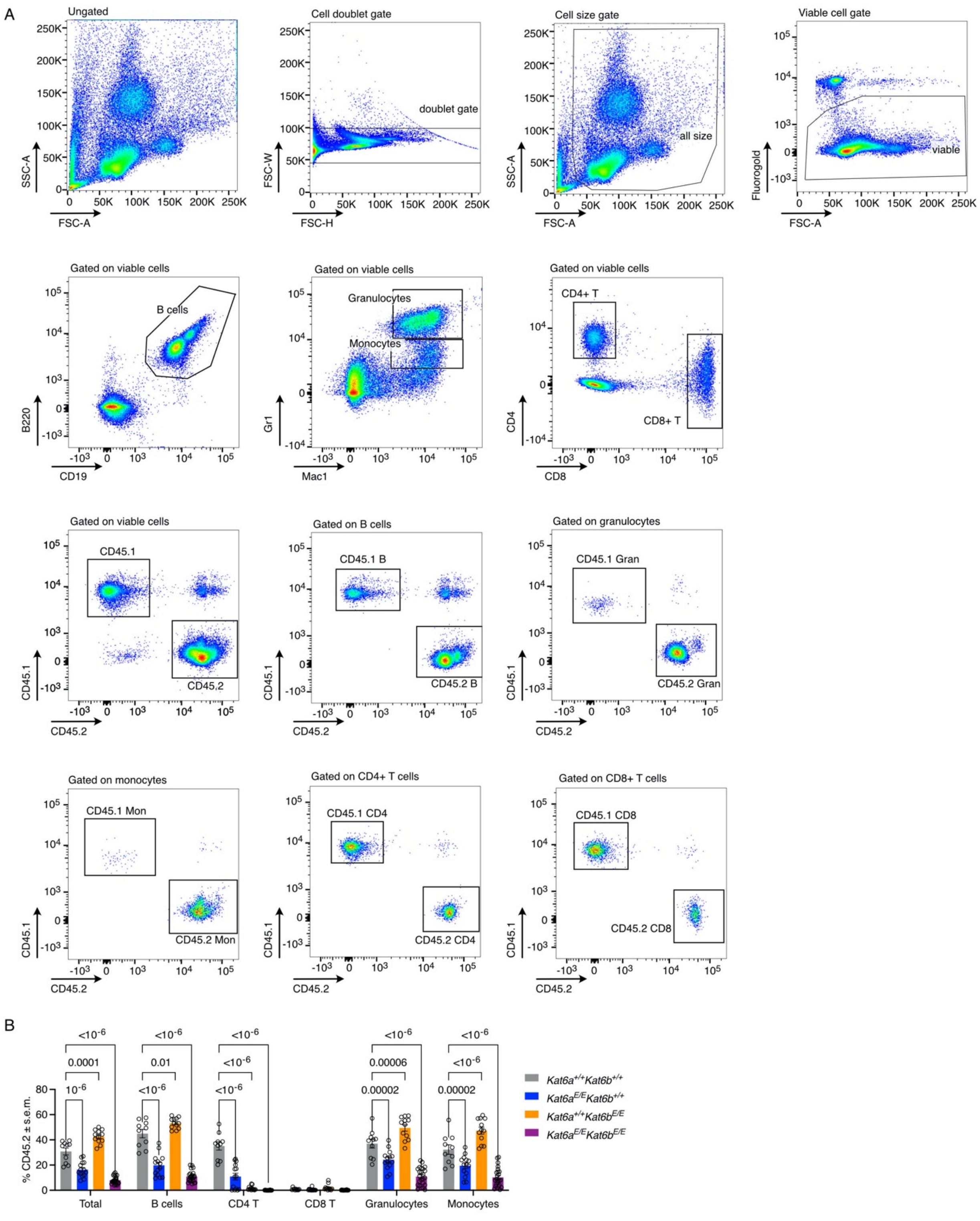

Development • Supplementary information

Fig. S4. Gating strategy and results of primary haematopoietic transplantation experiments – peripheral white blood cells.

Gating strategy for and results from the flow cytometry analysis of lethally irradiated recipient mice transplanted with E14.5 fetal liver haematopoietic cells of the following genotypes: *Kat6a*$^{+/+}$;*Kat6b*$^{+/+}$ (N = 10), *Kat6a*$^{E/E}$;*Kat6b*$^{+/+}$ (N = 14) *Kat6a*$^{+/+}$;*Kat6b*$^{E/E}$ (N= 12) and *Kat6a*$^{E/E}$;*Kat6b*$^{E/E}$ (N = 24). Donor (test) cells the cell surface marker CD45.2; transplant recipient cells and competitor cells express CD45.1.

(A) Gating strategy for the flow cytometric analysis of peripheral blood, separating leukocytes from test cell from competitor cells on the basis of the CD45.1 and CD45.2 cell surface markers.

(B) Percentage of donor cell (test cell) contribution (cell surface phenotype CD45.2) to peripheral blood B, T and myeloid cells 1 month after transplantation.

Data are presented as mean ± SEM calculated from the average value of the recipients receiving fetal liver cells of the same genotype. Each circle represents a single recipient (B). Data were analyzed by two-way-ANOVA with Tukey's multiple comparisons test (B).

Abbreviations and cell surface markers used to identify populations in Supplementary Table 3.

Development • Supplementary information

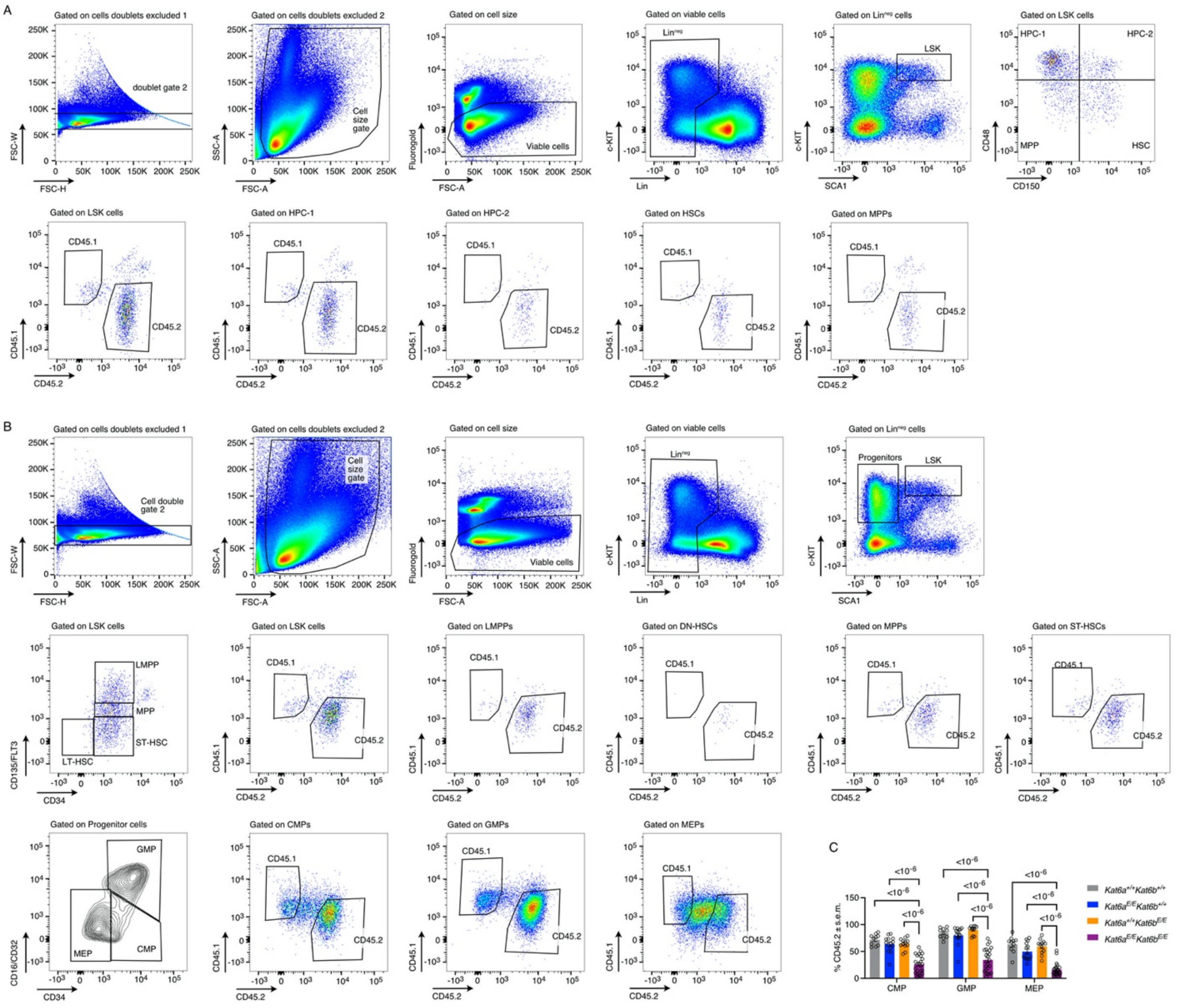

Fig. S5. Gating strategy and results of primary haematopoietic transplantation experiments – bone marrow stem and progenitor cells Gating strategy for and results from the flow cytometry analysis of lethally irradiated recipient mice transplanted with E14.5 fetal liver haematopoietic cells of the following genotypes: $Kat6a^{+/+}$;$Kat6b^{+/+}$ (N = 10), $Kat6a^{E/E}$;$Kat6b^{+/+}$ (N = 14) $Kat6a^{+/+}$;$Kat6b^{E/E}$ (N= 12) and $Kat6a^{E/E}$;$Kat6b^{E/E}$ (N = 24). Donor (test) cells the cell surface marker CD45.2; transplant recipient cells and competitor cells express CD45.1. (A,B) Gating strategy for the flow cytometric analysis of bone marrow haematopoietic stem and progenitor cells using SLAM markers (A) or CD135/FLT3 vs. CD34 (B) and CD16/CD32 vs.

CD34 to identify myeloid progenitors (B) and distinguishing test cell from competitor cells on the basis of the CD45.1 and CD45.2 cell surface markers.

(C) Percentage of donor cell (test cell) contribution (cell surface phenotype CD45.2) to myeloid progenitors in the bone marrow 16 weeks after transplantation.

Data are presented as mean ± SEM calculated from the average value of the recipients receiving fetal liver cells of the same genotype. Each circle represents a single recipient (C). Data were analyzed by two-way-ANOVA with Tukey's multiple comparisons test (C).

Abbreviations and cell surface markers used to identify populations in Supplementary Table 3.

Development • Supplementary information

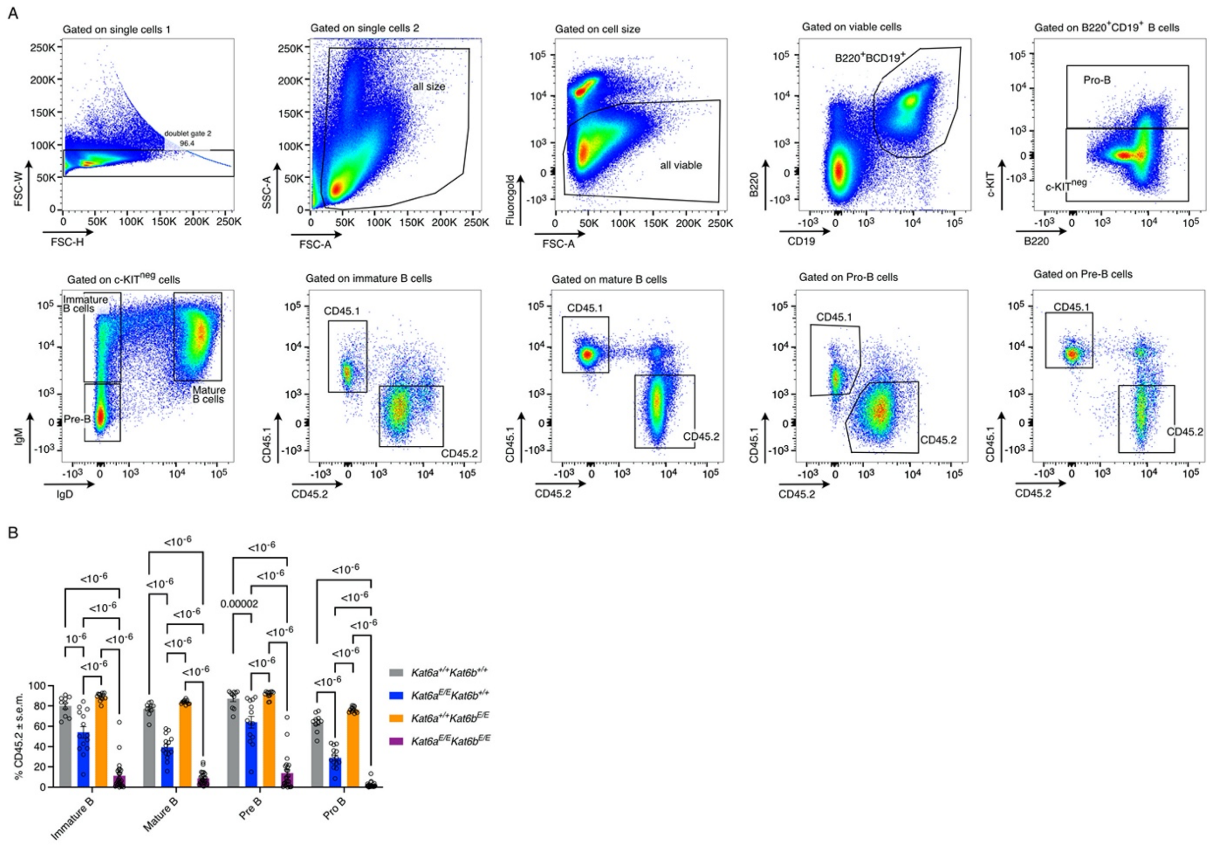

Fig. S6. Gating strategy and results of primary haematopoietic transplantation experiments – bone marrow B cell populations Gating strategy for and results from the flow cytometry analysis of lethally irradiated recipient mice transplanted with E14.5 fetal liver haematopoietic cells of the following genotypes: $Kat6a^{+/+}$;$Kat6b^{+/+}$ (N = 10), $Kat6a^{E/E}$;$Kat6b^{+/+}$ (N = 14) $Kat6a^{+/+}$;$Kat6b^{E/E}$ (N= 12) and $Kat6a^{E/E}$;$Kat6b^{E/E}$ (N = 24). Donor (test) cells the cell surface marker CD45.2; transplant recipient cells and competitor cells express CD45.1.

(A) Gating strategy for the flow cytometric analysis of bone marrow B cell progenitor cells.

(B) Percentage of donor cell (test cell) contribution (cell surface phenotype CD45.2) to B cell populations in the bone marrow 16 weeks after transplantation.

Data are presented as mean ± SEM calculated from the average value of the recipients receiving fetal liver cells of the same genotype. Each circle represents a single recipient (B). Data were analyzed by two-way-ANOVA with Tukey's multiple comparisons test (B). Abbreviations and cell surface markers used to identify populations in Supplementary Table 3.

Development • Supplementary information

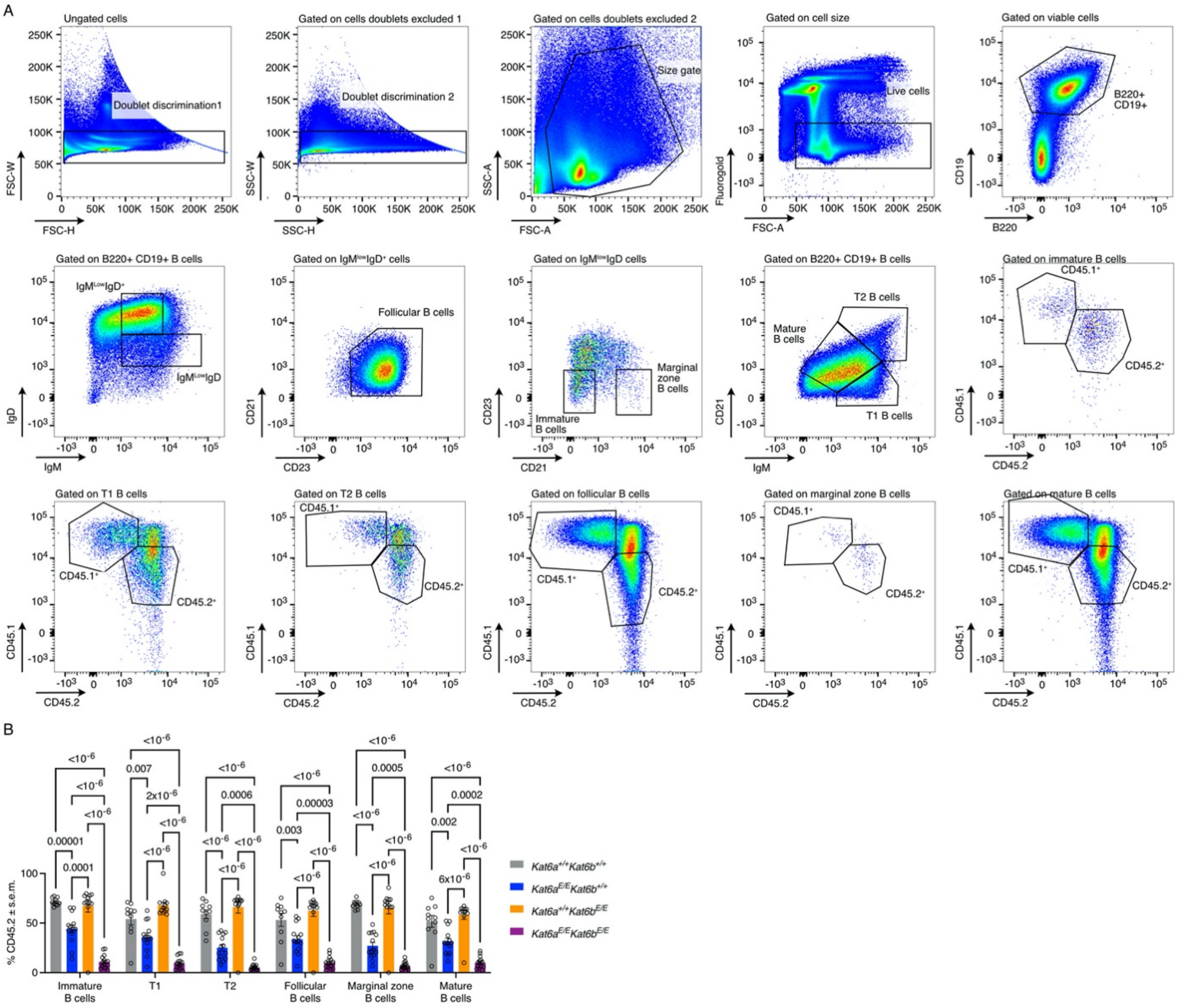

Fig. S7. Gating strategy and results of primary haematopoietic transplantation experiments –B cell populations in the spleen Gating strategy for and results from the flow cytometry analysis of lethally irradiated recipient mice transplanted with E14.5 fetal liver haematopoietic cells of the following genotypes: *Kat6a*$^{+/+}$;*Kat6b*$^{+/+}$ (N = 10), *Kat6a*$^{E/E}$;*Kat6b*$^{+/+}$ (N = 14) *Kat6a*$^{+/+}$;*Kat6b*$^{E/E}$ (N= 12) and *Kat6a*$^{E/E}$;*Kat6b*$^{E/E}$ (N = 24). Donor (test) cells the cell surface marker CD45.2; transplant recipient cells and competitor cells express CD45.1.

(A) Gating strategy for the flow cytometric analysis of spleen progenitors and mature B cell populations.

(B) Percentage of donor cell (test cell) contribution (cell surface phenotype CD45.2) to B cell populations in the spleen 16 weeks after transplantation.

Data are presented as mean ± SEM calculated from the average value of the recipients receiving fetal liver cells of the same genotype. Each circle represents a single recipient (B). Data were analyzed by two-way-ANOVA with Tukey's multiple comparisons test (B).
Abbreviations and cell surface markers used to identify populations in Supplementary Table 3.

Development • Supplementary information

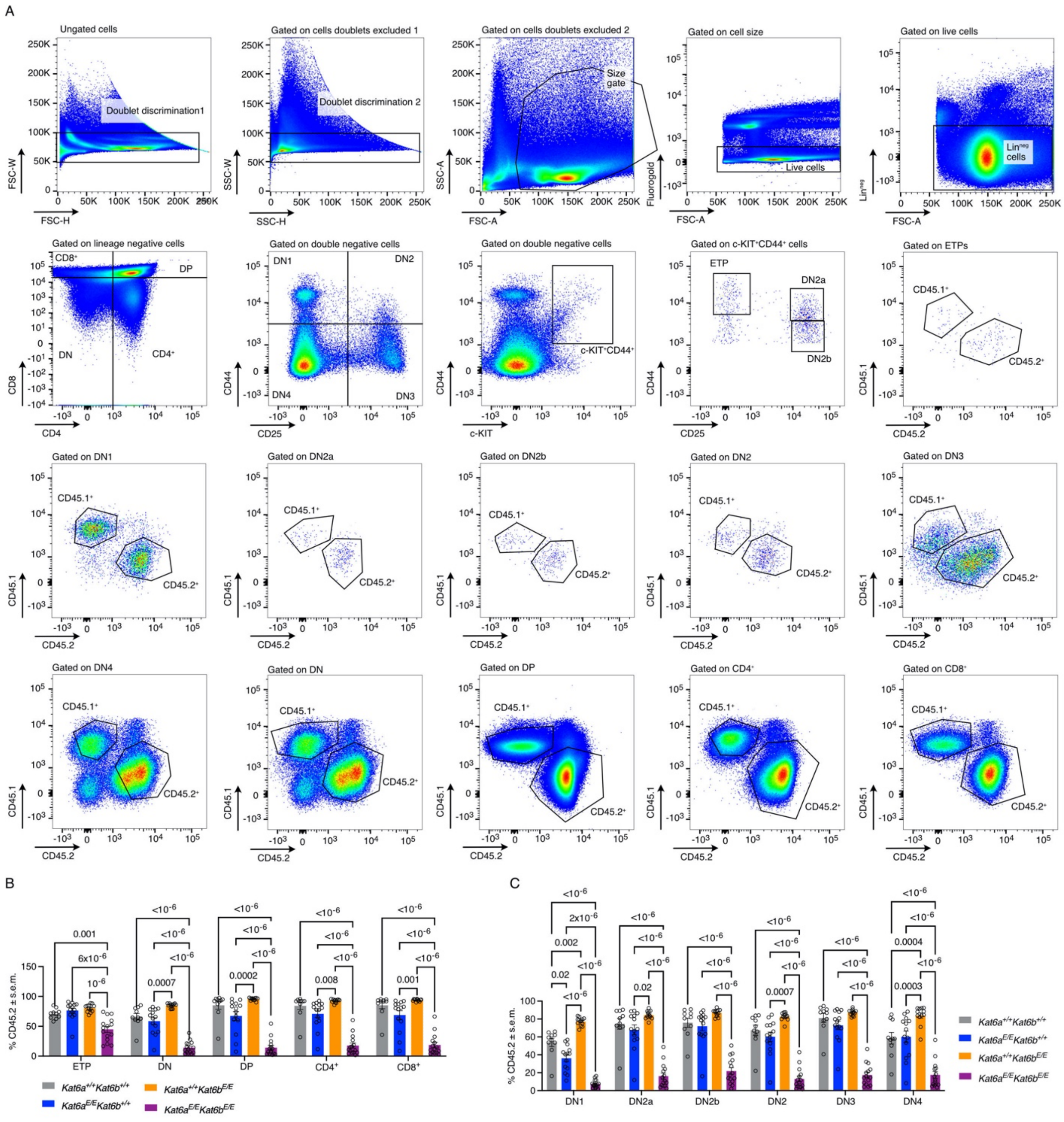

Fig. S8. Gating strategy and results of primary haematopoietic transplantation experiments – T cell populations in the thymus.

Gating strategy for and results from the flow cytometry analysis of lethally irradiated recipient mice transplanted with E14.5 fetal liver haematopoietic cells of the following genotypes: *Kat6a*$^{+/+}$;*Kat6b*$^{+/+}$ (N = 10), *Kat6a*$^{E/E}$;*Kat6b*$^{+/+}$ (N = 14) *Kat6a*$^{+/+}$;*Kat6b*$^{E/E}$ (N= 12) and

Kat6a$^{E/E}$;*Kat6b*$^{E/E}$ (N = 24). Donor (test) cells the cell surface marker CD45.2; transplant recipient cells and competitor cells express CD45.1.

(A) Gating strategy for the flow cytometric analysis of thymus progenitors and mature cells.

(B,C) Percentage of donor cell (test cell) contribution (cell surface phenotype CD45.2) to T cell populations in the thymus 16 weeks after transplantation.

Data are presented as mean ± SEM calculated from the average value of the recipients receiving fetal liver cells of the same genotype. Each circle represents a single recipient (B,C). Data were analyzed by two-way-ANOVA with Tukey's multiple comparisons test (B,C).

Abbreviations and cell surface markers used to identify populations in Supplementary Table 3.

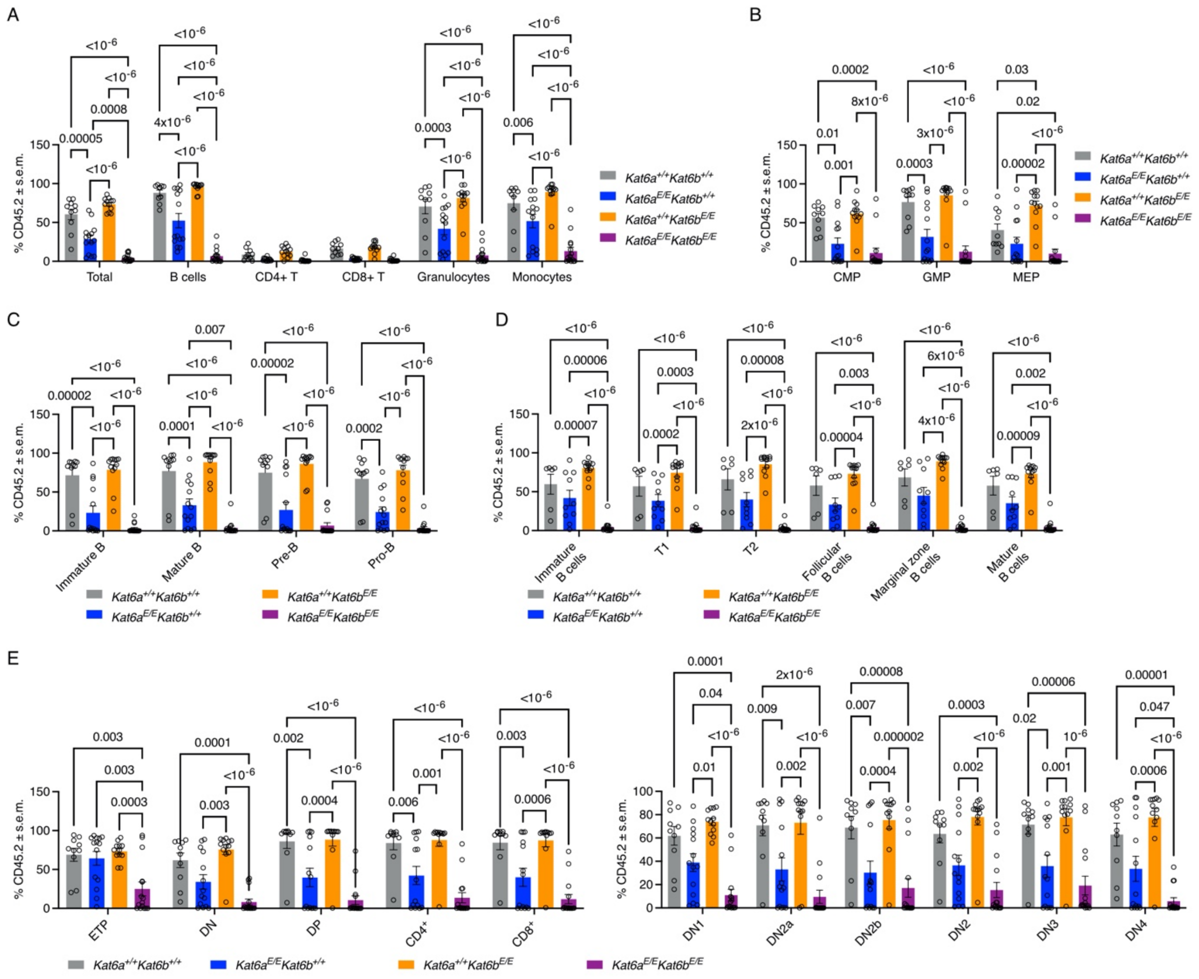

Fig. S9. Results of secondary haematopoietic transplantation experiments.

Flow cytometry analysis of lethally irradiated secondary recipient mice transplanted with bone marrow cells from the primary recipient of fetal liver haematopoietic cells of the following genotypes: *Kat6a*$^{+/+}$;*Kat6b*$^{+/+}$ (N = 10), *Kat6a*$^{E/E}$;*Kat6b*$^{+/+}$ (N = 14) *Kat6a*$^{+/+}$;*Kat6b*$^{E/E}$ (N= 12) and *Kat6a*$^{E/E}$;*Kat6b*$^{E/E}$ (N = 24).

(A-E) Percentage of donor cell (test cell) contribution (cell surface phenotype CD45.2) to peripheral white blood cells 4 weeks after transplantation (A), and 16 weeks after transplantation to myeloid lineage progenitors in the bone marrow (B), bone marrow B cell progenitor populations (C), B cell populations in the spleen (D) and T cell populations in the thymus (E). Data are presented as mean ± SEM calculated from the average value of the recipients receiving fetal liver cells of the same genotype. Each circle represents a single recipient (A-E). Data were analyzed by two-way-ANOVA with Tukey's multiple comparisons test (A-E). Abbreviations and cell surface markers used to identify populations in Supplementary Table 3.

Development • Supplementary information

Table S1. genotyping primers.

PCR		Primers (5' - 3')
**KAT6A wild type**	KAT6aG656-WT	CCCAATACCAACGTAAGGGC
	KAR6aG656E-R	TGTGGTGAGAAATGGAGAGG
**KAT6A mutant**	KAR6aG656E-mut	CCCAATACCAACGTAAGG**AA***
	KAR6aG656E-R	TGTGGTGAGAAATGGAGAGG
**Kat6B wild type**	KAT6BG577E-WToligoF	CCCAGCACCAAAGGCAAGGA
	KAT6BG577E-WToligoR	AGTGGGTGTCAGGGAAACTG
**Kat6B mutant**	KAT6BG577E-MUToligoF2	CCCAGCACCAAAGGCAAG**AG***
	KAT6BG577E-WToligoR	AGTGGGTGTCAGGGAAACTG

*Bold type indicates nucleic acid changes used to effect G to E amino acid substitution.

Table S2. Genes differentially expressed in Kat6aG656E/G656EKat6bG577E/G577E vs. Kat6a$^{+/+}$Kat6b$^{+/+}$ E9.5 embryos

Available for download at
https://journals.biologists.com/dev/article-lookup/doi/10.1242/dev.205559#supplementary-data

Table S3. Genes differentially expressed in Kat6a$^{-/-}$Kat6b$^{+/+}$ vs. Kat6a$^{+/+}$Kat6b$^{+/+}$ E9.5 embryos

Available for download at
https://journals.biologists.com/dev/article-lookup/doi/10.1242/dev.205559#supplementary-data

Table S4. Genes differentially expressed in Kat6a$^{+/+}$Kat6b$^{-/-}$ vs. Kat6a$^{+/+}$Kat6b$^{+/+}$ E9.5 embryos

Available for download at
https://journals.biologists.com/dev/article-lookup/doi/10.1242/dev.205559#supplementary-data

Development • Supplementary information

Table S5. Genes differentially expressed in Kat6aG656E/G656EKat6b$^{+/+}$ vs. Kat6a$^{+/+}$ Kat6b$^{+/+}$ E9.5 embryos

Available for download at
https://journals.biologists.com/dev/article-lookup/doi/10.1242/dev.205559#supplementary-data

Table S6. Genes differentially expressed in Kat6a$^{+/+}$Kat6bG577E/G577E vs. Kat6a$^{+/+}$ Kat6b$^{+/+}$ E9.5 embryos

Available for download at
https://journals.biologists.com/dev/article-lookup/doi/10.1242/dev.205559#supplementary-data

Table S7. GO terms BP (biological process) of genes differentially expressed in Kat6a$^{-/-}$Kat6b$^{+/+}$ vs. Kat6a$^{+/+}$Kat6b$^{+/+}$ and in Kat6aG656E/G656EKat6bG577E/G577E vs. Kat6a$^{+/+}$Kat6b$^{+/+}$ E9.5 embryos

Available for download at
https://journals.biologists.com/dev/article-lookup/doi/10.1242/dev.205559#supplementary-data

Table S8. GO terms BP (biological process) of genes differentially expressed in Kat6a$^{-/-}$Kat6b$^{+/+}$ vs. Kat6a$^{+/+}$Kat6b$^{+/+}$ but not in Kat6aG656E/G656EKat6bG577E/G577E vs. Kat6a$^{+/+}$Kat6b$^{+/+}$ E9.5 embryos

Available for download at
https://journals.biologists.com/dev/article-lookup/doi/10.1242/dev.205559#supplementary-data

Table S9. GO terms BP (biological process) of genes differentially expressed in Kat6aG656E/G656EKat6bG577E/G577E vs. Kat6a$^{+/+}$Kat6b$^{+/+}$ but not in in Kat6a$^{-/-}$Kat6b$^{+/+}$ vs. Kat6a$^{+/+}$Kat6b$^{+/+}$ E9.5 embryos

Available for download at
https://journals.biologists.com/dev/article-lookup/doi/10.1242/dev.205559#supplementary-data

Table S10. GO terms BP (biological process) of genes differentially expressed in Kat6a$^{+/+}$Kat6b$^{-/-}$ vs. Kat6a$^{+/+}$Kat6b$^{+/+}$ and in Kat6aG656E/G656EKat6bG577E/G577E vs. Kat6a$^{+/+}$Kat6b$^{+/+}$ E9.5 embryos

Available for download at
https://journals.biologists.com/dev/article-lookup/doi/10.1242/dev.205559#supplementary-data

Table S11. GO terms BP (biological process) of genes differentially expressed in Kat6a$^{+/+}$Kat6b$^{-/-}$ vs. Kat6a$^{+/+}$Kat6b$^{+/+}$ but not in Kat6aG656E/G656EKat6bG577E/G577E vs. Kat6a$^{+/+}$Kat6b$^{+/+}$ E9.5 embryos

Available for download at
https://journals.biologists.com/dev/article-lookup/doi/10.1242/dev.205559#supplementary-data

Table S12. GO terms BP (biological process) of genes differentially expressed in Kat6aG656E/G656EKat6bG577E/G577E vs. Kat6a$^{+/+}$Kat6b$^{+/+}$ but not in Kat6a$^{+/+}$Kat6b$^{-/-}$ vs. Kat6a$^{+/+}$Kat6b$^{+/+}$ E9.5 embryos

Available for download at
https://journals.biologists.com/dev/article-lookup/doi/10.1242/dev.205559#supplementary-data

Table S13. Genes differentially expressed in Kat6aG656E/G656EKat6b$^{+/+}$ vs. Kat6a$^{+/+}$Kat6b$^{+/+}$ E14.5 foetal liver haematopoietic cells

Available for download at
https://journals.biologists.com/dev/article-lookup/doi/10.1242/dev.205559#supplementary-data

Table S14. Genes differentially expressed in Kat6a$^{+/+}$Kat6bG577E/G577E vs. Kat6a$^{+/+}$Kat6b$^{+/+}$ E14.5 foetal liver haematopoietic cells

Available for download at
https://journals.biologists.com/dev/article-lookup/doi/10.1242/dev.205559#supplementary-data

Table S15. Genes differentially expressed in Kat6aG656E/G656EKat6bG577E/G577E vs. Kat6a$^{+/+}$Kat6b$^{+/+}$ E14.5 foetal liver haematopoietic cells

Available for download at
https://journals.biologists.com/dev/article-lookup/doi/10.1242/dev.205559#supplementary-data

Development • Supplementary information

Table S16. List of cell surface markers used for the FACS analyses of hematopoietic cells

Category	Cell type	Marker	Tissue type
Stem cell compartment	LSK	Lineage-Sca1+c-KIT+	BM
	HPC1 SLAM	Lineage-Sca1+c-KIT+CD150-CD48+	
	HPC2 SLAM	Lineage-Sca1+c-KIT+CD150+CD48+	
	HSC SLAM	Lineage-Sca1+c-KIT+CD150+CD48-	
	MPP SLAM	Lineage-Sca1+c-KIT+CD150-CD48-	
	DN-HSC	Lineage- Sca1+c-KIT+CD34-Flt3-	
	ST-HSC	Lineage- Sca1+c-KIT+CD34+Flt3-	
	MPP	Lineage- Sca1+c-KIT+CD34+Flt3+	
Progenitor	Progenitor	Lineage-Sca1-c-KIT+	BM
	CMP	Lineage-Sca1-c-KIT+CD34+CD16/32-	
	GMP	Lineage-Sca1-c-KIT+CD34+CD16/32+	
	MEP	Lineage-Sca1-c-KIT+CD34-CD16/32-	
Myeloid	Myeloid	Gr1+Mac1+	BM
B cell lineage	Pro-B	B220+CD19+c-KIT+	BM
	Pre-B	B220+CD19+c-KIT-IgD-IgM-	
	Immature B	B220+CD19+c-KIT-IgD-IgM+	
	Mature B	B220+CD19+c-KIT-IgD+IgM+	
	Marginal zone B cells	B220+CD19+CD23-CD21high	Spleen
	T1 – immature	B220+CD19+IgM+CD21-	
	T2 – immature	B220+CD19+IgM+CD21+	
	Mature spleen B cells	B220+CD19+IgMmed/highCD21med	
T cell lineage	CD4P	B220-CD19-Mac1-Gr1-Ter119-CD4+CD8-	Thymus
	CD8P	B220-CD19-Mac1-Gr1-Ter119-CD4-CD8+	
	DN	B220-CD19-Mac1-Gr1-Ter119-CD4-CD8-	
	DN1	B220-CD19-Mac1-Gr1-Ter119-CD4-CD8-CD25-CD44+	

Development • Supplementary information

	DN2	B220-CD19-Mac1-Gr1-Ter119-CD4-CD8-CD25+CD44+	
	DN3	B220-CD19-Mac1-Gr1-Ter119-CD4-CD8-CD25+CD44-	
	DN4	B220-CD19-Mac1-Gr1-Ter119-CD4-CD8-CD25-CD44-	
	ETP	B220-CD19-Mac1-Gr1-Ter119-CD4-CD8-CD25-c-KIT+	
	DN2a	B220-CD19-Mac1-Gr1-Ter119-CD4-CD8-CD25+c-KIThigh	
	DN2b	B220-CD19-Mac1-Gr1-Ter119-CD4-CD8-CD25+c-KITmed	
Myeloid,	Myeloid	Gr1+Mac1+	Peripheral
B-cells	B-cell	B220+CD19+	blood
T-cells	CD4+T-cell	CD4+	
	CD8+T-cell	CD8+	

LSK, lineage negative (negative for B220, CD19, CD4, CD8, GR1, LYG6 and TER119), SCA1 positive and c-KIT positive population. DN-HSC, long-term repopulating HSCs, double negative (DN) for CD34/FLT3. HSC SLAM, HSC with long-term repopulating ability defined by signaling lymphocytic activation molecule (SLAM) markers (CD150-positive and CD48-negative). HPC-1, Hematopoietic progenitor cells 1 (Kiel et al., 2005). HPC-2, Hematopoietic progenitor cells 2 (Kiel et al., 2005). ST-HSC, short-term repopulating hematopoietic stem cell. MPP, multipotent progenitor. CMP, common myeloid progenitor. GMP, granulocyte macrophage progenitor. MEP, megakaryocyte and erythrocyte progenitor. T1, transition type 1 B cells. T2, transition type 2 B cells. DN, double negative. ETP, early T-cell precursors.

References

Bergamasco, M. I., Ranathunga, N., Abeysekera, W., Li-Wai-Suen, C. S. N., Garnham, A. L., Willis, S. N., McRae, H. M., Yang, Y., D'Amico, A., Di Rago, L., et al. (2024). The histone acetyltransferase KAT6B is required for hematopoietic stem cell development and function. *Stem Cell Reports* **19**, 469-485.

Kiel, M. J., Yilmaz, O. H., Iwashita, T., Yilmaz, O. H., Terhorst, C. and Morrison, S. J. (2005). SLAM family receptors distinguish hematopoietic stem and progenitor cells and reveal endothelial niches for stem cells. *Cell* **121**, 1109-1121.

Thomas, T., Voss, A. K., Chowdhury, K. and Gruss, P. (2000). Querkopf, a MYST family histone acetyltransferase, is required for normal cerebral cortex development. *Development* **127**, 2537-2548.
